# Computational Design of Novel Allosteric Inhibitors for *Plasmodium falciparum* DegP

**DOI:** 10.3390/molecules26092742

**Published:** 2021-05-07

**Authors:** Sadaf Shehzad, Rajan Pandey, Pawan Malhotra, Dinesh Gupta

**Affiliations:** 1 Translational Bioinformatics Group, International Centre for Genetic Engineering and Biotechnology, New Delhi 110067, India; sadafshehzad@icgeb.res.in (S.S.); rajan@icgeb.res.in (R.P.); 2 Malaria Biology Group, International Centre for Genetic Engineering and Biotechnology, New Delhi 110067, India; pawanm@icgeb.res.in

**Keywords:** *Plasmodium falciparum*, serine protease, DegP, drug-design, bio-isosteric replacement, in silico screening

## Abstract

The serine protease, DegP exhibits proteolytic and chaperone activities, essential for cellular protein quality control and normal cell development in eukaryotes. The *P. falciparum* DegP is essential for the parasite survival and required to combat the oscillating thermal stress conditions during the infection, protein quality checks and protein homeostasis in the extra-cytoplasmic compartments, thereby establishing it as a potential target for drug development against malaria. Previous studies have shown that diisopropyl fluorophosphate (DFP) and the peptide SPMFKGV inhibit *E. coli* DegP protease activity. To identify novel potential inhibitors specific to PfDegP allosteric and the catalytic binding sites, we performed a high throughput in silico screening using Malaria Box, Pathogen Box, Maybridge library, ChEMBL library and the library of FDA approved compounds. The screening helped identify five best binders that showed high affinity to PfDegP allosteric (T0873, T2823, T2801, RJC02337, CD00811) and the catalytic binding site (T0078L, T1524, T2328, BTB11534 and 552691). Further, molecular dynamics simulation analysis revealed RJC02337, BTB11534 as the best hits forming a stable complex. WaterMap and electrostatic complementarity were used to evaluate the novel bio-isosteric chemotypes of RJC02337, that led to the identification of 231 chemotypes that exhibited better binding affinity. Further analysis of the top 5 chemotypes, based on better binding affinity, revealed that the addition of electron donors like nitrogen and sulphur to the side chains of butanoate group are more favoured than the backbone of butanoate group. In a nutshell, the present study helps identify novel, potent and *Plasmodium* specific inhibitors, using high throughput in silico screening and bio-isosteric replacement, which may be experimentally validated.

## 1. Introduction

Eukaryotic cells have developed a sophisticated system of proteases and molecular chaperones to regulate or dispose off unfolded/aggregated proteins, which works on the basic principle of recognizing the hydrophobic stretches of polypeptides [1,2]. This system attempts to refold defective proteins to the functional native structure; however, in the case of irreversibly damaged proteins, the polypeptides are subjected to proteolytic action [1]. The heat shock protein, DegP, a serine peptidase, also known as “HtrA” or “Protease Do” is involved in the degradation of non-native proteins in the periplasmic compartment for the quality control of cell [3,4]. Some DegP redirect themselves as chaperones to avoid protein aggregation formation, leading to serious health hazards like amyloid diseases [5]. DegP is evolutionarily conserved from bacteria, fungi, plants to mammals and perform its functional activity without utilizing energy currency, ATP [1]. DegP has been shown to facilitate the survival of the pathogens in adverse and stressful conditions [6], however, in eukaryotes, its function is diverse and not limited to protein quality control as they extend their action to regulate cell death, cell signaling and motility, maintaining homeostasis and other processes [7].

Since the last decade, many studies have been carried out deciphering the role of DegP. In Gram-negative bacteria such as *Salmonella enterica* serovar Typhimurium, *Brucella abortus, Brucella melitensis, Yersinia enterocolitica, Pseudomonas aeruginosa* and Gram-positive bacteria viz., *Streptococcus pyogenes, Staphylococcus aureus* and *Enterococcus faecalis*, DegP has shown to be essential for virulence factors [8], in *E. coli* it plays a role in outer membrane proteins (OMPs) biogenesis [9]. However, in *Campylobacter jejuni* it is known to induce host cell apoptosis and immunopathology during infection, and in its close relative *Helicobacter pylori*, it is an essential bifunctional gene with crucial intracellular and extracellular functions in cleaving E-cadherin [10]. In *Vibrio cholera*, it causes biofilm disruption and colonization defects [11]. In the case of mammalian HtrAs, its role is multi-faceted as is involved in various biological processes viz., embryogenesis, growth, apoptosis, neurodegenerative and neuromuscular disorder, cancer, act as modulators of programmed cell death, and in the enhancement of cytotoxic effects of conventional chemotherapy [12].

### DegP Structure and Mechanism

The DegP, belongs to trypsin clan SA, with a catalytic “His-Asp-Ser” triad [7]. DegP has been shown to have an extra-cytoplasmic compartment localization. The primary domain architecture of DegP consists of three distinct domains which include an N-terminal region known to have regulatory functions, a conserved trypsin-like protease domain (PD) and one or two PDZ domain (abbreviation derived from proteins enduring the repeats like PSD-95: mammalian postsynaptic density of 95 kDa, DLG: Drosophila discs large tumor suppressor and ZO-1: zonula occludens 1) at the C terminus [7]. PDZ domain is involved in mediating specific protein-protein interactions using 3–4 C-terminal residues, which vary depending on the interacting protein, providing a range of PDZ binding specificities [13]. The PfDegP organizes itself into complex, multimeric assemblies capable of switching between chaperone and protease, thereby functioning dually as protein repair and protein degradation machinery, in a temperature-dependent manner [14].

**Figure 1:**
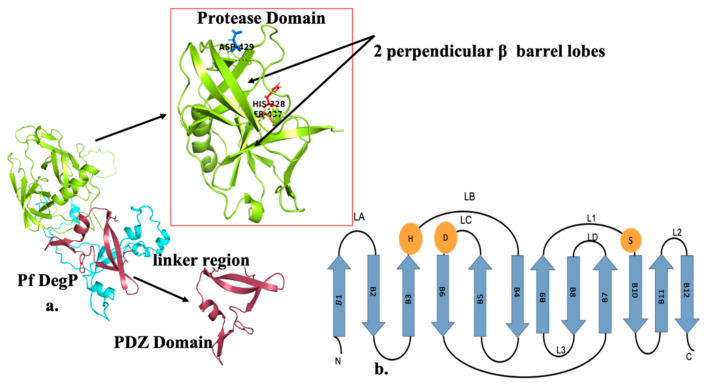
(**a**) Domain organisation and representation of PfDegP protein: Domain architecture of DegP consists of three distinct domains which include an N-terminal region known to have regulatory functions, a conserved trypsin-like protease domain (PD) shown in lemon having two perpendicular β barrel lobes and along with the catalytic triad displayed as sticks: Histidine (His 328) shown in red color, Aspartate (Asp 429) shown in marine, Serine (Ser 437) depicted in orange, Linker Region shown in cyan and one PDZ domain in brick red (**b**) Secondary structure elements of the DegP protease domain. The protease domain depicts a chymotrypsin fold and the B strands (shown as arrows) forming N and C terminal B barrels. For the sake of simplicity, the alpha helices are not depicted here. Catalytic triad residues: H, D and S are shown as orange spheres. Based on the chymotrypsin nomenclature, the loops of the protease domain are named, loops LD, L1, L2 and L3 shown in the figure are known to be regulatory.

The protease domain is known to have two perpendicular β-barrel lobes, encompassing the catalytic triad, with a C-terminal helix, as shown in Figure 1. The loops located at the C-terminal of the β-barrel lobes are known to participate in the active site’s assembly. The core of the protease domain is highly conserved [15]. The surface loops L1, L2 and L3 are known to play an important role in the adjustment of the catalytic triad. In chaperone formation, the loop LA interacts with loops L1 and L2 resulting in loop triad, LA*–L1–L2, which distorts the proteolytic site concerning catalytic triad formation, blocking the substrate-binding cleft, and abolishing the oxyanion hole and the S1 specificity pocket. Thus, in chaperone DegP’s protease domain is in an inactive state, thereby preventing the substrate binding and catalysis [16]. However, the PDZ domains exhibit structural similarity with the bacterial PDZ domains [17], analogous to other PDZ domains PDZ1 and/orPDZ2 should be involved in substrate binding. The β strand 14 in complex with the carboxylate binding loop and helix h of PDZ1 forms a deep binding cleft for the substrate [18]. Glu–Leu–Gly–Ile motif (GLGI) or Gly-Leu-Gly-Phe (GLGF) motif forms a highly positively charged region that localizes the carboxylate binding loop [13]. Among the PDZ domains, the carboxylate-binding loop (R/K-XXX-GLGF) is highly conserved, however, the second and fourth residues of the GLGI/GLGF motif are found to be always hydrophobic. The second glycine residue is conserved, but a serine, threonine, or proline replaces the first glycine in a few PDZs [13]. The LA*–L1–L2 loop triad is known to undergo a rearrangement, which may be due to the collapse of hydrophobic LA platform and enlargement of hydrophobic contacts at elevated temperatures, to provide active serine protease conformation for substrate binding [1].

**Figure 2:**
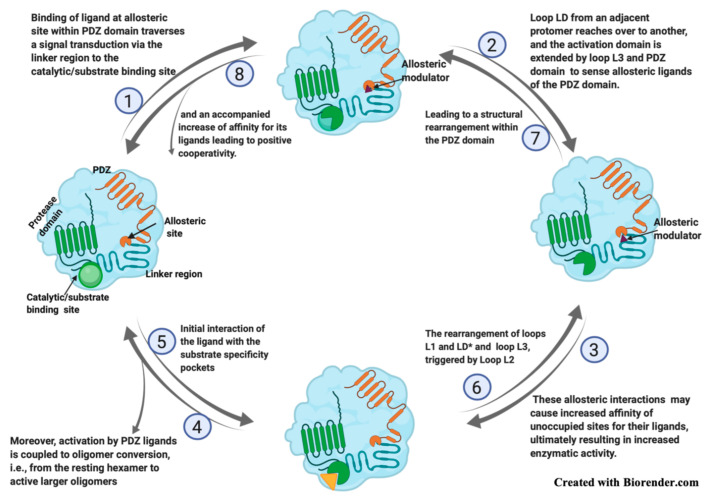
The interplay between the catalytic and allosteric sites in DegP. The structural rearrangement of substrate-binding pockets, proper positioning of the catalytic triads, and formation of the oxyanion holes occur via allosteric signal transduction across protomers resulting in structurally connected active sites and, ultimately in a positively cooperative manner.

The protease domains make the PfDegP chaperone’s sidewall, whereas the entry/exit gates are formed by PDZ domains which compared to molecular cages are wide. Furthermore, PfDegP in chaperone form consists of a distorted and inactive proteolytic site, thereby abolishing the substrate binding and catalysis [19]. The activity of complex enzymes like PfDegP is precisely regulated by a sophisticated oligomerization, cooperativity and allostery. DegP is known to have two binding sites per protomer, the catalytic site and the peptide-binding site of the PDZ domain, linked by an allosteric circuit. The activation domains (consisting of the regulatory loops as described earlier) are shared among the adjacent protomers mediating the coordinated activation of the catalytic sites [20]. The binding of ligands at the allosteric site channels a signal, opening the entry/exit gates, transferring a conformational change to the catalytic site to cause an activity modulation of the protein and increased substrate degradation (Figure 2).

The regulation of DegP is more complex as it requires loop LD from an adjacent protomer, and the activation domain is extended by loop L3 and protease domain to sense allosteric ligands of the PDZ domain. Also, PDZ ligands lead to activation and conversion to higher-order states from the resting hexamer to active larger oligomers [21]. However, the strong ligand binding to the catalytic site causes the same mechanistic flow, but in reverse order, i.e., ligand bind to the catalytic site and the binding of which causes the loop L2 to trigger the rearrangement of loops L1, L3 and LD*. This leads to structural rearrangement within the PDZ domain and leads to the increased affinity of the PDZ domain for its ligands, suggesting the high concentration of inhibitor mimics the effect of allosteric ligands of the PDZ domain, leading to the formation of oligomers from hexamers, leading to a positive cooperativity [21].

We have shown the essentiality of PfDegP for malarial parasite’s survival [6]. However, the DegP homologs are present only in *Plasmodium* species infecting primates viz., *P. falciparum, P. vivax, P. ovale, P. malariae, P. knowlesi*. *P. falciparum* DegP (PfDegP, Pf3D7_0807700) is expressed during the sporozoite, trophozoite and schizont- stages within the parasite [6]. In-vivo complementation studies with *E. coli* null mutants exhibited 2.5-fold induction in an episode of febrile temperature and complements the growth defects of the *E. coli* in DegP knockout temperature-sensitive strain JW0157 (JWDdegP) [6]. PfDegP has serine protease activity, as shown in in vitro protease activity assays [6]. The interacting partners of PfDegP are elucidated to be *P. falciparum* heat shock protein (PfHsp70) and *P. falciparum* enolase (PfEno) as revealed by the co-immunoprecipitation experiments [6]. In terms of its ability to survive and combat the thermal stress condition and essentiality in the parasite’s life cycle at the intraerythrocytic stage makes PfDegP as potential drug target [6]. With the recent advancement in bioinformatics, high throughput in silico drug screening, better pharmacological design tools and faster computing have made the process of drug discovery comparatively cost-effective and less time-consuming. In this study, we have discussed the structural insights of PfDegP and performed high throughput drug screening for the identification of specific lead compounds within the two binding sites S1 and S2, S1 being allosteric in nature and S2 as catalytically active. Five potential PfDegP specific hits from docking studies of both the sites were identified and their mechanism of interaction is discussed. In addition to this, molecular dynamics (MD) simulations and a statistical thermodynamic analysis of water molecules were used to explain the important molecular interactions for a series of substituted potential hits, post high-throughput screening.

## 2. Result

### 2.1. Conserved Domain, Evolutionary and Interlog Analysis of PfDegP

PlasmoDB [22] and conserved domain database (CDD) [23] analysis on PfDegP shows that PfDegP belongs to serine protease family having a trypsin-like serine protease domain (275–483 amino acids), a PDZ domain (630–695 amino acids) and presence of a signal sequence (1–28 amino acids) (Figure S1). The multiple sequence alignment analysis [24] for the whole length protein for *P. falciparum* (Pf), *P. vivax* (Pv), *E. coli* (Ec), *T. gondii* (Tg), *A. thaliana* (At) and *H. sapiens* (Hs) revealed conservation for the protease catalytic triad region (Figure S2). The analysis further revealed presence of Gly-Ser-Gly-Phe (GSGF) motif except in *T. gondii* (STGF) within the protease domain before catalytic triad rather than within the PDZ domain. The Glu–Leu–Gly–Ile motif (GLGI) or Gly-Leu-Gly-Phe (GLGF), known to form a highly positively charged region localizing the carboxylate binding loop was partially present in *P. falciparum* (Figure S2). The earlier studies provide support to our findings as the second of the two glycines is highly conserved, but the first glycine of the motif may be replaced by serine, threonine, or proline. However, for the conserved Leu of GLGF loop present in the PDZ domain of E. coli, Leu residue was not observed in the *Plasmodium* PDZ domain region although the 2nd Gly and Ile were conserved in all studied organisms.

The ortholog analysis for PfDegP protein using the OrthoMCL database (OG5_133046) shows 1–4 protein isoforms in the studied model organisms (Table S1) [25]. Most of the apicomplexans encode a single copy of DegP protease whereas, in plants, 3 copies of DegP proteins were found to be encoded. Among apicomplexans, *Toxoplasma gondii* and *Neospora caninum* encode two copies of DegP protease whereas the maximum copy number of four was found in *Ricinus communis*, a perennial flowering plant species. This suggests that during evolution, DegP protein may have undergone multiple duplication events resulting in multiple copies of DegP isoforms to perform specific functions. Our analysis could not find direct PfDegP orthologs in the host *Homo sapiens* and other mammals. The phylogenetic analysis performed to study evolutionary relationship revealed a high degree of clustering for orthologs from apicomplexan with a single copy of DegP protease into a single cluster with evolutionary closeness with free-living unicellular, colonial flagellate (*Monosiga brevicollis*), soil-dwelling amoeba (*Dictyostelium discoideum*), and small sea anemone (*Nematostella vectensis*). In general, the evolutionary tree can be grouped into two large clusters, one with apicomplexans and the other consisting of algae and plant species (Figure S3). However, for *T. gondii* and *N. caninum* encoding two copies of DegP protein, the second isoform protein displayed clustering into the group that included plants and algae. Free-living unicellular and colonial flagellate (*Monosiga brevicollis*), which is considered the closest living relatives to animals, forms the connecting link between these two-broad clusters of plants and apicomplexan. Further to identify the closest homolog of PfDegP in Homo sapiens and other mammal species, a PSI-BLAST search was conducted to search for the nearest homolog. The most similar human protein is a HtrA1 (DegP) protease, 3NZI with ~22% query coverage and ~33% identity (Table S2). All the protease homologs identified in mammals were DegP protease which shares low identity and coverage only within the protease domain, therefore not directly included in the PfDegP ortholog analysis (average percentage identity of 44.7%).

The STRING database analysis to identify potential interlogs of PfDegP revealed that PfDegP interacts primarily with IMP-specific 5′-nucleotidase, α-tubulin N-acetyl transferase, rhomboid protein 9, acyl-CoA synthetase and other proteins as mentioned in S1D [26]. The proteins predicted to interact with PfDegP, are involved directly or indirectly in the detoxification, apoptosis regulation and maintenance of homeostasis thereby, suggesting that PfDegP plays a crucial role in parasite survival (Figure S4).

### 2.2. Determination of Binding Sites within the 3D Structure of PfDegP

Due to the absence of any PfDegP crystal structure, we have used an in-silico approach to generate the 3D PfDegP structure. The secondary structure consists of 27 α-helices and 25 β-pleated-sheets as predicted by PSI PRED (Figure S5) [27]. The closest PDB template which showed 42.80% similarity and 45% sequence coverage (not covering the entire protease domain) was 5ILN_A (*Arabidopsis thaliana* protease DO like-9). Therefore, we used I- TASSER to predict full-length PfDegP 3D structure (Figure S6) [28]. The predicted structure had TM-scores, RMSD and C-score of 0.35 + −0.12, 17.0 + −2.8 and −1.83 respectively. The number of decoys used during the generation of best model was 172. The RMSD analysis based on molecular dynamics suggested the stability and correctness of the predicted model (Figure S7) [29,30]. Further, the Ramachandran plot of the predicted model showed 93.5% of residues in the allowed region (Figure S8) [31]. ERRAT score for the predicted model was 85.930, verifying the model’s overall quality (Figure S9). The domain architecture of the predicted PfDegP protein is shown in Figure 1.

Next, Fpocket analysis to identify binding pockets present in PfDegP, predicted 53 binding pockets for PfDegP, which were compared with the active site of the template protein. Pocket S1 includes ILE294, SER(331, 419, 746), ALA327, HIS(328, 356, 546), ASP(359, 541,543), ASN(420, 699, 741), GLY(490, 530), VAL(491, 694), TYR696, LYS(695,745, 747), LEU697, GLN535, GLU749 residues with a pocket binding score of 60.442, drugability score of 0.167, hydrophobicity score of 18.679, polarity score of 49 and a charge score of 6. The predicted pocket also consists of HIS328, one residue out of a known catalytic triad of PfDegP. However, the second predicted pocket S2 consists of PHE(116, 206, 207, 212, 316, 371, 379, 669), SER(119, 215, 471), ASN(200, 230, 243), ARG(202),VAL(203,374,447),LEU(204,213,322,377,475,477),PRO(205),HIS(214,218,321,378,472,478), THR(217, 468), LYS(220,221, 228, 368, 479), ASP(222, 372, 373), MET(224), ALA(225, 376), ILE(317, 318, 477), GLU(319, 667), GLY(320), TYR(375), with a score of 37.636, drugability score of 0.221, hydrophobicity score of 22.588 with a charge score of 0.00. Thus, the two binding sites identified in PfDegP, the catalytic site comprising of the His 328 residues from the catalytic triad, while the allosteric site encompasses the regulatory loops/activation domain. The binding of ligands at the catalytic and allosteric sites was studied.

**Figure 3:**
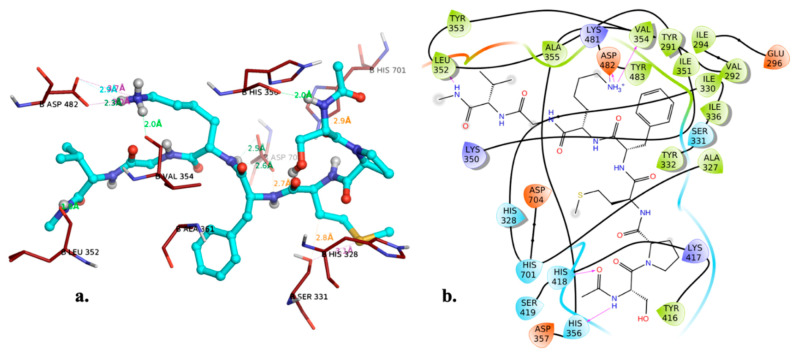
(**a**) Docked pose of peptide inhibitor in the least biding energy conformation along with the interacting residues (**b**) the interaction diagram of PfDegP with peptide inhibitor.

**Figure 4:**
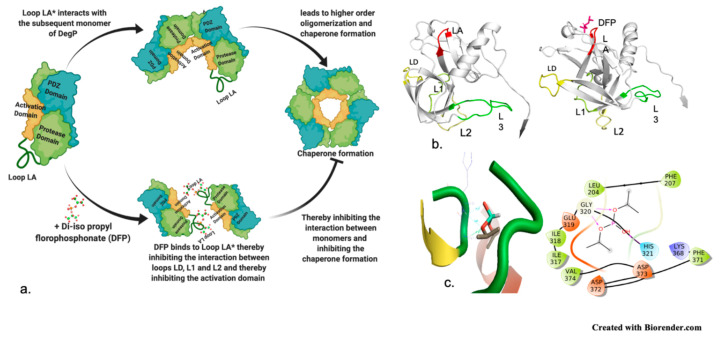
PfDegP activation domains in bound conformation with DFP, a mechanism-based inhibitor of DegP (**a**): Mechanism of Action of Di-iso Propyl fluorophosphate in synapse acting on acetylcholinesterase and inhibiting its function of breakdown of choline esters by phosphorylating the crucial serine residue (**b**) cell homeostasis by inhibiting the serine protease DegP by interacting with loop LA (regulatory loop) and inhibiting the activation domain (L1-L2-LD) (**c**) Docked pose of DFP with PfDegP in the least binding energy conformation along with the interacting residues, and interaction diagram of DFP, mechanism-based inhibitor.

The substrate derived inhibitor peptide SPMFKGV and the mechanistic inhibitor DFP (diisopropyl phosphonate) both were used as reference compounds [32]. It was quite interesting to note that the peptide inhibitor exhibited interaction with the catalytic His 328 thus exhibiting its binding within the protomer’s active site pocket as it the lacks the C-terminal necessary to binding with the Fpocket predicted “pocket 0” (Figure 3a,b), however, the small compound libraries were docked based on the binding pocket of the peptide. The residues of the predicted “pocket 1” overlap with the binding site residues of the template, additionally, when the mechanism-based inhibitor DFP (di-isopropyl phosphonate) was subjected to dock the PfDegP by encompassing the entire protein within the docking grid, it was quite interesting to observe that the DFP irrespective of having a catalytic residue HIS328 within the pocket0 docked within the “pocket 1” and exhibited a covalent interaction with the HIS321 (Figure 4a–c).

To further validate this, different binding conformations of the PfDegP-DFP complex was analysed, but none of the docked confirmations showed involvement of any residues from the catalytic triad. Further analysis of PfDegP-DFP complex revealed that HIS321 localized in loop LA of the basic trypsin protease domain of serine protease family (Figure 4c) [33,34]. The previous reports suggest that loop LA (also referred as a regulatory loop) is the key for forming higher-order oligomers and in DegP6 closely interacts with the active site loops LD, L1 and L2 of the opposing trimeric ring and the ligand binding to the protease site [35]. Therefore, it may be elucidated that the binding of DFP to HIS321 of loop LA may disrupt interactions between loops LD, L1 and L2 and thereby inhibit the DegP protease. DFP, the mechanism-based inhibitor, yet retained its inhibition mechanism as in the case of E. coli DegP [35], thus, suggesting that though this protein evolved and is in the eukaryotic system yet shows the coherence in inhibition mechanism with that of the bacterial system (Figure 4a,b). We hypothesized that blocking the activation domain of PfDegP by small-molecule may allosterically inhibit the signal parsing and therefore, may act as an inhibitor and may offer potential therapeutic value.

To check binding affinity of DFP with predicted closest PfDegP homolog in human, 3NZI, docking study was performed using a similar protocol as discussed for PfDegP. The docking analysis showed a different binding site for human DegP as compared to PfDegP. Further, the analysis revealed that all ligands that bind to human DegP do not interact with the residues present in the protease catalytic active sites or any residues present in the linker region (Figures S10 and S11).

### 2.3. Allosteric Site 

To identify potent *Plasmodium*-specific inhibitors with no to very less side effects on the host, we used publicly available compound datasets, most of them already tested on various cell cultures with a known target and inhibitory efficacy like ChEMBL-NTD database, Maybridge library, malaria box, MMV Pathogen Box, and FDA Approved library. ChEMBL-NTD (26,800 compounds), Maybridge compounds (14,400 compounds), malaria box (400 compounds) and the MMV Pathogen Box (400 compounds) provide open access to phenotypic screening datasets of relevance to neglected tropical diseases and malaria. Additionally, FDA approved library (1561 compounds) was also screened in search of repurposing of existing drugs. The compounds were filtered based on the docking score instead of the structure-based virtual screening method. The receptor grid was generated with the same amino acid residues as predicted in pocket 1. All the libraries were docked in Flare using Cresset [36]. 

**Figure 5:**
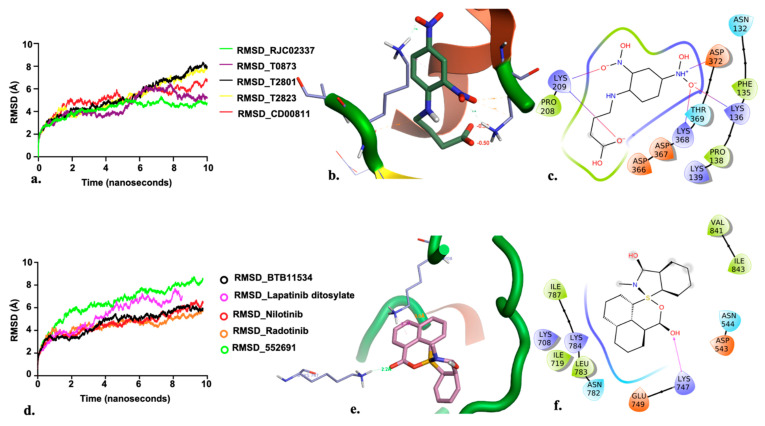
(**a**) RMSD for the top five PfDegP-complexes from the allosteric site and it was quite evident that the two complexes RJC02337 and T0873 attained stability. The complex PfDegP-RJC02337 (green) was quite stable for the whole simulation period exhibiting the least fluctuations, whereas complex PfDegP-T0873 (purple) tends to attain its stability after a simulation period of ~8 nanoseconds. However, the complexes PfDegP-T2823 (black), PfDegP-T2801 (yellow) and CD00811 (red) exhibited a continuous increase in their RMSD, indicating their unstable interaction with the protein. (**b**) Least binding energy pose of Maybridge_RJC02337 docked with PfDegP (**c**) Ligand interaction diagram of Maybridge_RJC02337 showing the interacting residues with PfDegP (**d**) The RMSD for the top five PfDegP- complexes and it was quite evident that the DegP complex with BTB11534, Lapatinib ditosylate, Radotinib, 552691 attained stability while the complex with Nilotinib exhibited the increased RMSD (**e**) Least binding energy pose of Maybridge_BTB11534 docked with PfDegP (**f**) Ligand interaction diagram of Maybridge_BTB11534 showing the interacting residues with PfDegP.

Docked poses for each ligand were analysed in order of ascending Lead Finder Rank Score (LF Rank Score), providing the best score to the correct (experimentally observed) ligand pose [37]. The lower (more negative) is the LF Rank Score, the higher is the likelihood that the docked pose reproduces the crystallographic pose. Maybridge_RJC02337, {4-(2,4-Dinitroanilino)butanoate} exhibited the LF Rank Score of −16.742, the binding energy of −8.149 kcal/mole. Ligand interaction diagram revealed the H-bond interaction with LYS 209, LYS 368 and hydrophobic interactions with PHE135, ASN132, PRO138, ASP366, ASP367 (Figure 5. The compounds T0873 (dinitolmide), T2823 (crocin), T2801 (aristolochic acid) and the compound Maybridge_CD00811 (*N*-[(*Z*)-furan-2-ylmethylideneamino]-5-methyl-2,4-dinitroaniline) showed the LF Rank Score of ~−15. The binding energy and the details of violation of the rule of 5 of the top five leads and the DFP are tabulated in Table 1. The interaction details of the compounds are presented in Figures S12 and S13. The previous published literature analysis for the identified hits revealed that compounds T0873, T2823 and T2801 are known to show different pharmacological activities. The compound T0873 exerts coccidiostatic activity against *Eimeria tenella* and *Eimeria necatrix* by arresting the asexual developmental stages of the parasite life-cycle [38]. Similarly, T2823 has been investigated in the treatment of cisplatin-induced hepatotoxicity via TLR4/NF-κBp50 signalling and BAMBI modulation of TGF-β activity [39]. T2801 plays multiple role as a nephrotoxin, a carcinogenic agent, a mutagen, a toxin and a metabolite too [40,41,42,43,44] (available experimental data regarding the other biological activities of the compounds and the activity profile is shown in Table S3). 

### 2.4. Catalytic Site Binders

**Figure 6:**
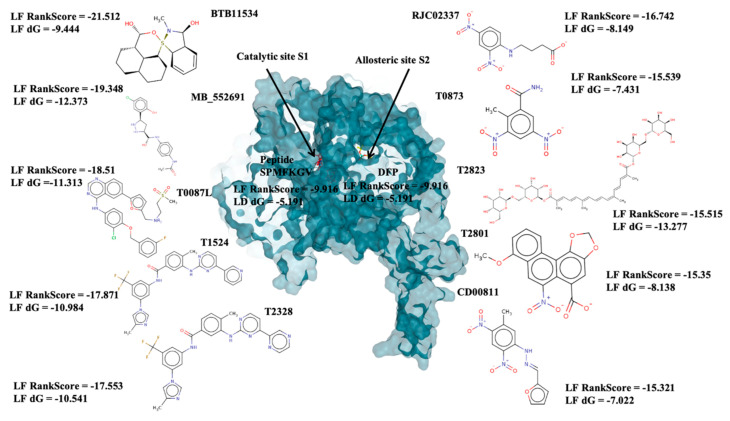
The depiction of the allosteric and catalytic pocket of PfDegP in bound confirmation with DFP and peptide SPMFKGV and the top 5 screened compounds of both the pockets with their binding score (LF Rank Score) and binding affinity (LFdG).

With respect to the binding site S1, the compounds Maybridge_BTB11534 exhibited the least LF Rank Score of −21.512 and the binding energy LFdG −9.444 kcal/mole, however, the compound was found to have a single hydrogen bond with Lys 747 and exhibited hydrophobic interactions with ASN 782, LYS 708, LYS 784, ILE843, GLU749. The other compounds of the top 5 were T0078L-lapatinib ditosylate monohydrate, T1524-nilotinib, T2328-Radotinib and malaria box compound with Chembl id 552,691 exhibiting the LF Rank Score value from −19 to −16.495 while the binding energy in the range of −12 to −10 kcal/mol (Figure 5 and Figure 6). The details of the other top four compounds of the pocket S1 in their docked conformation are shown in Figures S14 and S15. The top five compounds from each pocket (S1), Smiles formatted structures and other parameters are tabulated in Table 2. Similar to the allosteric site screened compounds, published literature analysis revealed that compounds T0078L, T1524 and T2328 inhibit other biological targets, specifically proteins involved in cancer. T0078L is a known inhibitor of several tyrosine kinase receptors involved in tumor cell growth and is currently used in the therapy of advanced breast cancer and other solid tumors [45,46]. T1524 has been indicated to treat newly diagnosed Philadelphia chromosome positive chronic myelogenous leukaemia (CML) in the chronic phase [47,48,49,50]. The role of T2328 has also been studied for the treatment of different types of cancer viz., Philadelphia chromosome-positive (Ph+) chronic myeloid leukemia (CML), and its role as Bcr-Abl tyrosine-kinase inhibitor [51,52,53]. The experimentally validated non-PfDegP targets and their activities are mentioned in Table S4.

The protein-ligand complex stability, binding mode and potential interactions within the binding site of PfDegP were analysed for the top five compounds by molecular dynamic simulation studies using AMBER 18 (Flare, Cresset package) [36,37,54,55]. All the complexes were equilibrated for 1.1 ns and then subjected to 10 ns at 300 K. The complex stability was analysed by the root mean squared deviation plot (RMSD) and compared before and after simulation. Frames were captured every 1ps and saved a trajectory, a total of 1000 frames were generated during the simulation of each complex. MD simulations were performed using the default parameters, charges of ligands binding to both the allosteric site and the catalytic site were set using GAFF and AMI-BCC methods [55,56]. In terms of the allosteric site, the three complexes were stable except the compound T2823 and T2801 based on the analysis of RMSD after MD simulation (Figure 5). Based on the MD representative structure of PfDegP complexes, per-residue energy decomposition based on the Molecular Mechanics Generalised Based Surface Area (MM-GBSA) method using the standalone version of AMBER 12 (Table S5) revealed stable protein-ligand interaction for our best hit PfDegP-RJC02337 [57,58]. The parameters used during analysis are discussed in Appendix A.

### 2.5. Compound Annotations and Design of New Chemotypes

#### 2.5.1. Absolute Binding Free Energy Calculation of Allosteric Modulator

**Figure 7:**
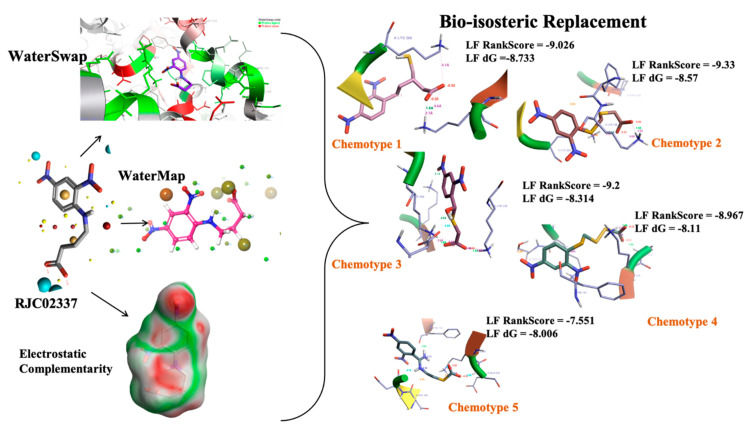
The 2D structure of compound RJC02337, WaterSwap analysis of PfDegP with compound RJC02337: Residues Lys368, Lys350, Lys139, Lys142, Arg141, Lys88, Pro138, Lys136, Lys209 and Lys481(shown in green sticks) exhibits the favourable contributions in free binding energy to PfDegP-RJC02337 interaction, however, the residues Asp373, Asp372, Asp366, Asn132, Glu210, Asp132 are the residues which are exhibited to negatively contribute to the protein-ligand binding interaction within the same PfDegP-RJC02337 complex; Watermap of RJC02337, predicting the site 17, 84, 104 and 93 exhibiting the high delta G of 4.35, 2.71, 2.19 and 2.57; Electrostatic complementarity of RJC02337 to protein; the docked pose of the compound exhibiting the least binding energy as chemotype 1: exhibiting the binding energy of −8.733 kcal/mol docked and the interacting residues with the protein PfDegP based on templating with the compound RJC02337, chemotype 2: exhibiting the binding energy of −8.57 kcal/mol docked with the protein PfDegP along with the interacting residues, chemotype 3: showing the binding energy of −8.314 kcal/mol docked with the protein PfDegP, chemotype 4: exhibiting the binding energy of −8.11 kcal/mol docked with the protein PfDegP, chemotype 5: interacting residues, exhibiting the binding energy of −8.006 kcal/mol docked with the protein PfDegP.

We performed WaterSwap analysis for our best hit, PfDegP-RJC02337 (Figure 7) to know the favourable residues involved in the binding interaction. The analysis revealed that residues Lys368, Lys350, Lys139, Lys142, Arg141, Lys88, Pro138, Lys136, Lys209 and Lys481 are determined to be the ones providing favourable contributions in free binding energy and Asp373, Asp372, Asp366, Asn132, Glu210, Asp132 are the residues which provided an unfavourable contribution to the protein-ligand binding interaction. 

#### 2.5.2. Novel Chemotypes Identification for Compound RJC02337 Based on Watermap, Electrostatic Complementarity and Bioisosteric Replacement

Based on WaterSwap analysis results, we performed Watermap analysis using the PfDegP-RJC02337 complex to identify potential modification sites within the compound RJC02337 to design potent inhibitors specific to the PfDegP. The Watermap analysis predicted multiple easily displaceable high energy water, which were not displaced by RJC02337 compounds. The results identified the site; 17, 84, 104 and 93 with high ΔG of 4.35, 2.71, 2.19 and 2.57, respectively (Figure 7). For compound RJC02337, modifications adjacent to the above-stated sites may provide an opportunity in achieving the improved potency of the compound. Also, the electrostatic complementarity of the inhibitor to the protein was visualized to have a better understanding of the dynamics being offered by the inhibitor as electrostatic interactions act as the key contributor to the enthalpic component of the binding free energy ΔG. Important insights can be gathered by assessing the electrostatic match between ligands and binding pockets, which provides insight to improvise the selectivity and pharmacokinetic parameters to understand SAR and design novel potent target specific compounds.

The electrostatic complementarity revealed that compound RJC02337 in a docked pose with PfDegP can be classified into three regions: a region with no electrostatic contribution (white color), the region depicting the perfect electrostatic clash (red color) and the region with perfect electrostatic complementarity (green color) (Figure 7). The compound RJC02337 along the butanoate group was selected for bio-isosteric replacements against the 31 databases offered by Spark package. Bio-isosteric replacement addresses the limitations of failed metabolism and pharmacokinetics while still retaining the potency and efficacy of the initial lead compound. The structural changes to the lead compound using bio-isosteres alter the compound’s size, electronic distribution, shape, polarizability, polarity, dipole, lipophilicity, and pKa, while still retaining potent target engagement. Therefore, for the rational modification of a lead compound, the bio-isosteric approach was used to have a more attractive therapeutic agent with improved potency, selectivity, metabolic and toxicological properties. Bio-isosteric replacement is an integral part of conventional drug design by applying replacements to initial hits, increasing the probability of their chemotypes for successfully entering the trials. The replacements offered in modified compounds were then redocked based on templating with PfDegP-RJC02337 complex using Flare, the compounds which exhibited the least LF dG score compared to RJC02337 on redocking were found to be 231 in number (Table S6). The compounds which exhibited the least LF dG were visualized and had contributed to the better understanding and development of the pharmacophore prototype. The top 5 compounds are shown in Table 3. The results exhibited that the addition of the nitrogen group or sulphur group within the butanoate group of RJC02337, enhances the inhibiting potential without disturbing target specificity. The addition of thiol group to the butanoate decreases the binding energy by approximately 3-fold thereby exhibiting the least binding energy of −8.733kcal/mol as compared to the compound RJC02337 exhibiting the energy of −5.519 kcal/mol, however, the chemotype 2 and chemotype 3 exhibits least binding energy of −8.57 and −8.314, respectively, where the carbon of the butanoate was replaced by sulphur thereby proposing the hypothesis that the sulphur group is favoured in the sidechain rather as a part of the main carbon chain also that two sulphur groups offer better binding energy as compared to one sulphur atom. In terms of carbon of butanoate being replaced by a sulphur atom, the number of sulphur atoms needs to be optimum as two being more favoured than the one or three sulphur atoms in number as exhibited in chemotype 4. However, there is only a slight variation in the binding energy by all the chemotypes discussed above. The interacting residues of the chemotypes have been shown in Figure 7 and Figure S16.

## 3. Discussion

**Figure 8:**
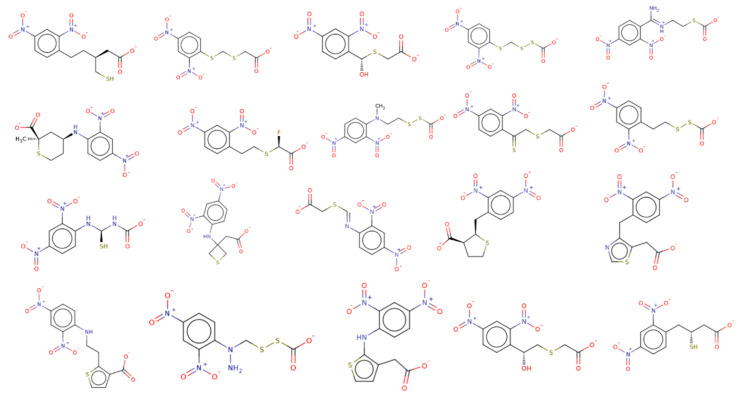
The top 20 chemotypes derived from the parent RJC02337 from Bio-isosteric replacement.

This work was a continuation of our efforts to identify potent inhibition of human malaria parasite *Plasmodium falciparum*, PfDegP. This knowledge is needed for a better understanding of the PfDegP inhibitory/ligand binding mechanism. As the PfDegP consists of two binding sites and may involve the allosteric mechanism of regulation. Based on the results obtained from docking the inhibitors at both the sites, two structurally varied inhibitors are identified RJC02337 and BTB11534 as shown in Figure 5 as both the binding sites are dynamically different as predicted by F pocket. The site S1 (catalytic) is more polar and charged as compared to the site S2 (allosteric), as is also evident that the ligand exhibiting least LF Rank Score compliments the binding site as the inhibitor RJC02337 is more polar and charged due to the presence of Nitrogen and Oxygen atoms as compared to BTB11534. As allosteric site leads to the activation mechanism of PfDegP we tried to explore the dynamics offered by the pocket and the inhibitor docked with high binding affinity to further design and construct the chemotypes. The electrostatic complementarity to protein, WaterSwap and Watermap studies as discussed in Figure 7 were conducted in order to explore the interactions which can be modified and the groups and the linker to be explored for Bio-isosteric replacements. The 30 databases offered by Spark software were explored and the chemotypes obtained were then template based docked to PfDegP. 231 chemotypes are identified which exhibited the better binding affinity as compared to parent molecule RJC02337. The 2D structures of the top 20 resulted chemotypes are displayed in Figure 8. Majority of the top compounds were known to possess the Sulphur moiety either within the backbone or within the heterocyclic aromatic ring leading to the hypothesis that the binding site prefers the electronegative atoms.

## 4. Materials and Methods

### 4.1. Conserved Domain, Evolutionary and Interlog Analysis

The PfDegP sequence (ID:PF3D7_0807700) was retrieved from PlasmoDB (release 36) [22]. Conserved domains were annotated using the Conserved Domain Database (CDD) search [23]. OrthoMCL (version 5) database was used to identify *P. falciparum* orthologs [25]. Multiple sequence alignment was performed for the retrieved sequences, using ClustalW using default parameters [24]. A Phylogenetic tree was inferred using MEGA X [59]. Sequence alignment was performed using MUSCLE and the tree was constructed employing the Neighbour Joining method [60] as the statistical method and inferred using 1000 bootstrap replicates [61]. Missing data was processed using partial deletion, with 95% site coverage cut-off. The evolutionary distances were computed using the JTT matrix-based method [62] and are in the units of the number of amino acid substitutions per site. The phylogenetic tree was generated using iTOL version 5.6 [63]. STRING database (version 11.0) was used to identify PfDegP interactions using an organism-specific search [64]. Cytoscape v3.7 was used to visualize the protein-protein interactions and the network was analysed using a network analyser taking into consideration the betweenness centrality, closeness centrality and the degree with a cut-off value of 4 [26].

### 4.2. Generation of PfDegP 3D Structure and Its MD Simulation

The PfDegP secondary structure was predicted using PSIPRED [27]. The PfDegP 3D structure was generated by I-TASSER (Iterative Threading ASSembly Refinement) [28]. MD simulation was performed for further optimization, validation, and downstream analysis of the predicted 3D model. GROMACS (v4.6) with CHARMM27 [29] force field was used to perform MD simulation in an aqueous environment [30]. The quality of the simulated protein structure was verified using the Ramachandran Plot generated by RAMPAGE (http://mordred.bioc.cam.ac.uk/~rapper/rampage.php; accessed on 17 March 2020) and ERRAT [31].

### 4.3. Binding Site Analysis

F-pocket [65] was used to predict binding pockets present in the simulated PfDegP model. F-pocket employs the determination of the whole ensemble of alpha spheres from the protein structure in the first step returning a pre-filtered collection of spheres. In addition to this, it identifies clusters of spheres close together to predict pockets, thereby removing clusters of poor interest. Finally, calculating properties from the atoms of the pocket and scoring them. The binding sites predicted by I-TASSER were also considered [28].

### 4.4. Virtual Screening and MD Trajectory Analysis

DegP is known to be inhibited by peptides and Di-isopropyl fluorophosphate (DFP) in *E*. *coli* [35]. So, the refined PfDegP model was energy minimized and prepared using AutoDock (AD) tools [66]. The docking grid was set up enclosing the entire protein structure to identify the best docking pose and the interacting residues to define the best binding site. In addition to this, DegP activity inhibition by peptide SPMFKGV [32] was performed using Glide. The docking grid was set up taking the centroid of the ligand and the protein employing the MM-GBSA scoring method treating the receptor as a rigid structure, while the active site potentials were softened to simulate small adjustments of the receptor to the ligand. The peptide was treated flexible [67].

The compound libraries (Malaria Box, Pathogen Box, Maybridge library and ChEMBL library), except the library of FDA approved compounds were filtered using Lipinski’s rule of 5. The screened compounds were docked into the explicit binding site of the refined PfDegP structure using Flare and the ligands were selected based on the LF Rank Score [36,37]. Lower (more negative) the Rank Score, higher is the docked pose’s likelihood to reproduce the crystallographic pose. Protein-ligand complex was subjected to Molecular Dynamics Simulation using Flare (Cresset package) and free binding energy calculations using standalone version of AMBER 12 [54]. Protocol details have been provided in Appendix A1. Three-dimensional visualization of the structures was generated using PyMOL. Interaction diagrams were generated using Schrödinger Suite. 2D structure visualization was created using smi2Depict (https://re.edugen.wiley.com/cgibin/Smi2DepictWeb.py; accessed on 20 March 2021).

### 4.5. Absolute Binding Free Energy Calculation

The absolute binding free energy was calculated using WaterSwap from Flare. WaterSwap uses condensed-phase simulations to calculate the absolute protein-ligand binding free energies. The analysis was run with the default parameters and normal calculation method using the AMBER force field. The WaterSwap provides the consensus free binding energies calculated by different methods calculated from weighted arithmetic mean of Bennett (50%), TI (30%), and FEP (20%) free energy estimators, and the associated error (Maximum less Minimum score of the free energy estimators). The parameters used are discussed in Appendix A2. Monte Carlo (MC) sampling is used to verify the absolute binding free energy [68].

### 4.6. Hydration Energy Calculations

WaterMap [69], a molecular dynamics-based method was used for prediction of the locations, enthalpy (ΔHsolv), and entropy (−TΔSsolv) of water molecules relative to the bulk medium in the solvation layer of proteins, based on the theory of inhomogeneous solvation of Lazaridus and Karplus [70], which takes into account the enthalpy directly from nonbonded interactions and entropy to be computed from a local expansion of spatial and orientational correlation functions [69,71]. Desmond32 (OPLS_2005 force field [72,73] was used to perform MD simulations. TIP4P water molecules in an orthorhombic box extending 10 Å from the protein surface were used for protein solvation. Neutralization of the total charge of the system was carried out by the addition of sodium and chloride ions to the water box. To prevent the entry of the ions to the solvent analysis region during the simulation, a unidirectional harmonic restraint potential was applied to them, however, the ion beyond the region of analysis was freely mobile. Desmond relaxation protocol was applied in WaterMap with default parameters involving successive stages of minimization and heating, followed by production MD simulation of 2.0 ns with positional restraints (5 kcal mol^−1^Å^2^) on the heavy protein atoms. WaterMap was operated from Maestro, the ligands were picked for the protein PfDegP and the waters within 10 Å of the selected residues were analyzed, with the default parameters. WaterMap calculates the energies pertaining to the equilibrium thermodynamic stability of water in the binary protein water system. Transfer of water from solvation sites occupied by the ligand to bulk medium and from bulk medium to all vacant solvation sites are caused by the association and dissociation of the ligand. Water transfer energies (WT_H_, WT_S_, and WT_G_) were calculated from WaterMap energies. Assumptions made comprises that the transfer of water molecules from the solvation layer to the bulk medium are independent during the ligand association based on their degree of spatial overlaps with a ligand molecule. However, in some cases, there may be the co-existence of non-overlapped water molecules with bound ligands and could form H-bond bridges while in other instances, spatial positions of solvating water molecules may shift in the presence of a ligand at the expense of energy [74] the parameters are discussed in Appendix A3. Based on the ratio of the centre-to-centre distance between a given ligand atom and a neighbouring solvation site divided by the sum of the van der Waals radius of that atom and the solvation site radius (overlaps assumed to exist for ratios >0.5), the overlaps between each docked inhibitor and solvation sites were evaluated. Each overlapped solvation site was also used to evaluate the entropic contributions (WT_S_) irrespective of the polarity of the over-lapping inhibitor atoms as the total entropy released from the transfer of favourable solvation to a bulk medium is always strictly favourable, and contributes solely to ΔG_dissoc_ [74].

### 4.7. Electrostatic Complementarity Analysis and Bio-Isosteric Replacements in Anti-Malarial Drug Design

Electrostatic complementarity analysis was carried out using Flare, using the default parameters [75]. To obtain a truly novel core scaffold, performed bio-isosteric replacement with Spark (Cresset, UK) [37,76].

## 5. Conclusions

PfDegP, a serine protease, has been shown to play a crucial role in felicitating the parasite survival and growth. In the study, we applied multiple Computer aided drug designing tools to identify and design PfDegP specific inhibitors. The evolutionary analysis revealed that PfDegP is evolutionary conserved but shares low similarity with the mammalian proteases, however, the catalytic triad exhibits highly conserved nature. Further, we explored the PfDegP inhibitory mechanism using a reference compound DFP and peptide SPMFKGV, as shown for *E. coli* DegP. The high throughput in silico screening coupled with molecular dynamics simulations helped in the identification of PfDegP specific compound RJC02337, 4-(2,4-Dinitroanilino)butanoate and BTB11534. Bio-isosteric replacement study was performed taking into consideration the results obtained from WaterSwap, WaterMap and electrostatic complementarity analysis in order to design the chemotypes for the compound RJC02337 which may be comparatively more potent without altering the specificity. The analysis demonstrated that a simple addition of the thiol group or a nitrogen group as a side chain exhibits better binding affinity than the compound RJC02337 without disturbing the target specificity. The chemotype 1 with the thiol group in the side chain is more favoured than the changes offered in the butanoate carbon chain. In addition to this, an optimum number of sulphur atoms are required for favourable binding interactions. In summary, the present study helps identify PfDegP specific potent inhibitors using in silico approaches, which have potential therapeutic value for human malaria parasite that can be further validated using in vitro/in vivo experiments.

## Figures and Tables

**Table 1:** The docking score and binding energy of the top five compounds docked against PfDegP within the allosteric pocket (S2).

Structure	2D Structure	Title	LF Rank Score ^1^	LF dG ^2^	LF vs. Score ^3^	MW ^4^	Atoms	SlogP ^5^	TPSA ^6^	RB ^7^	Rof5 ^8^
[O-][N+](=O)c1cc([N+]([O-])=O)ccc1NCCCC([O-])=O	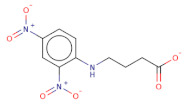	RJC02337	−16.742	−8.149	−9.451	268.2	19	1.6	143.8	7	0
O=C(N)c1cc([N+]([O-])=O)cc([N+]([O-])=O)c1C	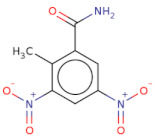	T0873	−15.539	−7.431	−9.133	225.2	16	0.7	134.7	3	0
O[C@@H]1[C@H](O[C@@H]([C@H](O)[C@@H]1O)CO)OC[C@@H]2[C@H](O)[C@H](O)[C@H](O)[C@H](O2)OC(=O)/C(=C/C=C/C(C)=C/C=C/C=C(\C=C\C=C(\C(O[C@@H]3[C@@H](O)[C@@H](O)[C@@H](O)[C@H](O3)CO[C@@H]4[C@@H](O)[C@@H](O)[C@@H](O)[C@H](O4)CO)=O)C)C)C	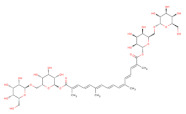	T2823	−15.515	−13.277	−19.591	977	68	−0.6	391.2	32	3
[O-][N+](=O)c1cc2c(OC)cccc2c3c4c(OCO4)cc(c13)C([O-])=O	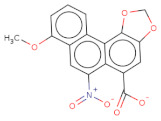	T2801	−15.35	−8.138	−10.132	340.3	25	3.2	113.6	3	0
[O-][N+](=O)c1c(cc(N/N=C/c2ccco2)c([N+]([O-])=O)c1)C	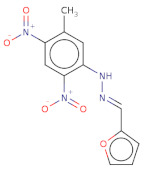	CD00811	−15.321	−7.022	−9.005	290.2	21	2.1	129.2	5	0
OP(OC(C)C)OC(C)C	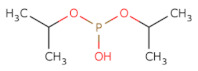	DFP	−9.916	−5.191	−5.268	165.1	10	2.5	35.5	4	0

^1^ This function is used for ranking ligand poses obtained during a docking run. The purpose of this function is to reproduce experimentally observed ligand poses as well as possible. ^2^ This scoring function has been designed to perform accurate estimation of the free energy of protein-ligand binding for a given protein-ligand complex. ^3^ This function has been designed to produce maximum efficiency in virtual screening experiments i.e., to assign higher scores to active ligands (true binders) and lower scores to inactive ligands. ^4^ Molecular weight. ^5^ Log of the octanol/water partition coefficient (including implicit hydrogens). ^6^ Polar surface area calculated using group contributions with the parameters of Ertl et al. (2000). ^7^ Rotatable bonds. ^8^ Lipinski’s rule of five.

**Table 2:** The docking score and binding energies of the top five compounds docked to the PfDegP catalytic pocket (S1).

Structure	2D Structure	Title	LF Rank Score	LF dG	LF vs. Score	MW	Atoms	SlogP	TPSA	RB	Rof5
O[C@H]1[C@H]2C=CC=C[C@H]2[S@]3(N1C)[C@H]4CCC[C@H]5CCC[C@@H]([C@H]54)[C@@H](O3)O	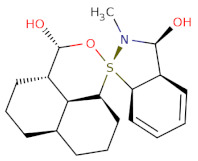	BTB11534	−21.512	−9.444	−10.711	351.5	24	3.4	52.9	2	0
Clc1ccc(O)c([C@H]2C[C@H](NN2)[C@@H](O)Nc3ccc(NC(=O)C)cc3)c1	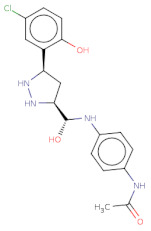	MB_552691	−19.348	−12.373	−13.444	376.8	26	2.7	105.6	7	1
Clc1cc(Nc2c3c(ncn2)ccc(c4ccc(o4)C[NH2+]CCS(=O)(=O)C)c3)ccc1OCc5cc(F)ccc5	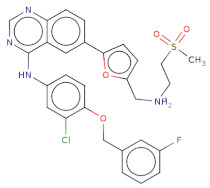	T0078L	−18.51	−11.313	−14.248	582.1	40	5.9	110.9	8	1
FC(F)(F)c1cc(NC(=O)c2ccc(c(Nc3nccc(n3)-c4cnccc4)c2)C)cc(-n5cnc(c5)C)c1	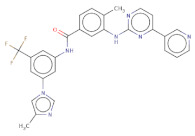	T1524	−17.871	−10984	−12.821	529.5	39	6.4	97.6	7	2
FC(F)(F)c1cc(NC(=O)c2ccc(c(Nc3nccc(n3)-c4cnccn4)c2)C)cc(-n5cnc(c5)C)c1	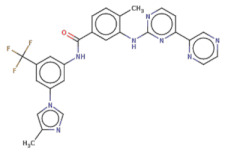	T2328	−17.553	−10.541	−12.76	530.5	39	5.8	110.5	7	2

**Table 3:** The modified top five compounds from RJC02337 {4-(2,4-Dinitroanilino) butanoate} obtained by bio-isosteric replacements, their structure and the binding energy.

Structure	2D Structure	MW	Atoms	Rof5	Attachment Point 1 Type	Attachment Point 2 Type	LF dG	LF vs. Score	LF Rank Score
SC[COOH](CCc1ccc([N+]([O-])=O)cc1[N+]([O-])=O)CC([O-])=O	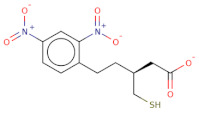	313.3	21	0	Csp3	Csp3	−8.733	−8.868	−9.026
[O-][N+](=O)c1cc([N+]([O-])=O)ccc1SCSCC([O-])=O	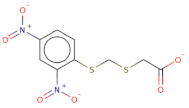	303.3	19	0	S	Csp3	−8.57	−8.757	−9.33
O[COOH](SCC([O-])=O)c1ccc([N+]([O-])=O)cc1[N+]([O-])=O	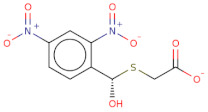	287.2	19	0	Csp3	Csp3	−8.314	−9.108	−9.2
[O-][N+](=O)c1cc([N+]([O-])=O)ccc1SCSSC([O-])=O	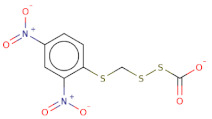	321.3	19	0	S	S	−8.11	−8.956	−8.967
[O-][N+](=O)c1cc([N+]([O-])=O)ccc1/C(=[NH+]/CCSC([O-])=O)N	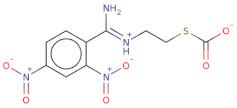	314.3	21	0	Csp2	S	−8.006	−7.295	−7.551

## Data Availability

The data presented in this study are available as supplementary materials.

## Supplementary Materials

The following are available online, Figure S1: Identification of conserved domains of PfDegP by CDD predicting a signal sequence (1–28 amino acids), trypsin-like serine protease domain (275–483 amino acids) belonging to serine protease family and a PDZ domain (630–695 amino acids), Figure S2: Multiple Sequence Alignment of PfDegP with selected organisms, Figure S3: Phylogenetic tree of PfDegP: The evolutionary history was inferred using the Neighbour-Joining method, Figure S4: Protein-protein interactions of PfDegP predicted by STRING v11.0 and visualized using Cytoscape v 3.7 and network analyser, Figure S5: Secondary structure prediction of PfDegP using PSI-PRED, Figure S6: (a) Predicted 3D structure of PfDegP using the template 4FLN_A (*Arabidopsis thaliana*, chloroplast Protease Do-like 2), (b) Superimposition of PfDegP homology model with the template (RMSD value of 0.203), Figure S7: (a) RMSD plot of PfDegP trajectories during molecular dynamics simulation of 10 ns (b) Radius of gyration of PfDegP, Figure S8: Ramachandran Plot of PfDegP after simulation of 20 nanosecond, Figure S9: ERRAT score of the PfDegP model, Figure S10: PfDegP (green in color) in docked pose with DFP (brick red in color) and human DegP, 3NZI (cyan in color) in docked pose with DFP (limon in color) exhibiting binding interactions in different pockets. The pocket residues are labelled in both 3NZI and PfDegP, Figure S11: PfDegP (green in color) in docked pose with T2801 (brick red in color) and human DegP, 3NZI (cyan in color) in docked pose with T2801 (limon in color) exhibiting binding interactions in different pockets. The pocket residues are labelled in both 3NZI and PfDegP, Figure S12: showing interaction of potential hits of allosteric site binders: (a) T0873 Dinitolmide, (b) T2823 Crocin, (c) T2801 Aristolochic Acid, (d) CD00811showing the residues involved in Hydrogen bonding and hydrophobic interactions, Figure S13: Interaction Diagrams of (a) T0873 Dinitolmide, (b) T2823 Crocin, (c) T2801 Aristolochic Acid, (d) CD00811 showing the residues involved in Hydrogen bonding and hydrophobic interactions, Figure S14: showing interaction of potential hits: (a) MB_552691, (b) T0087L, (c) T1524, (d) T2328 showing the residues involved in Hydrogen bonding and hydrophobic interactions, Figure S15: Ligand interaction diagrams: (a) MB_552691, (b) T0087L, (c) T1524, (d) T2328 showing the residues involved in Hydrogen bonding and hydrophobic interactions, Figure S16: Interaction diagram of (a) chemotype 1, (b) chemotype 2, (c) chemotype 3, (d) chemotype 4, (e) chemotype 5 showing the residues involved in Hydrogen bonding and hydrophobic interactions, Table S1: The orthologs of PfDegP retrieved from OrthoMCL DB having the OrthoMCL id OG5_133046, Table S2: Table depicting the percent identity and query coverage in model organisms, Table S3: The non PfDegP related biological activities of the shortlisted drug molecules, binding to PfDegP allosteric site; Table S4: The drug activity towards other biological targets of the compounds identified as PfDegP inhibitors binding at catalytic site; Table S5: MMGBSA result of PfDegP-2236 complex; Table S6: List of 231 chemotypes obtained after bio-isosteric replacement of RJC02337.

## Appendix A

### Appendix A.1. Binding Energy Calculation Using AMBER

Binding energies of the selected compounds were calculated using Generalized Born (GB) methods [77] in AMBER (ver. 12) [78]. AMBER LEaP using leaprc.ff99SB forcefield was used to prepare the input files for the simulation programs. The parameterization of small molecules (ligands) was done using GAFF by Antechamber. Energy minimization was done using AMBER12 Simulated Annealing with NMR-Derived Energy Restraints (SANDER). The equilibration of the system and the molecular production simulation was performed using PMEMD. All the MD simulations were performed with distance constraints using SHAKE on the hydrogen atoms, 2fs time step, and Langevin Dynamics for temperature control. A weak restraint (2.0 Kcal/mol-Å^2^) on the complex was used during energy minimization, heat and density equilibrium. A cut-off value of 8 Å was used during MD simulation. The complex was equilibrated for 1.1ns (500 ps, 500 ps and 1ns heat, density and pressure equilibrium respectively) before 10ns production simulation at 300K. Root mean squared deviation plot (RMSD) was generated before and after production run to check the stability of the complex. MMPBSA.py script was used to calculate the free binding energy of the protein–ligand complex and contribution of active pocket residues in binding [79]. The calculation of hydrogen bond occupancy and life-time for protein-ligand complex during molecular dynamics simulation was done using cpptraj. The distance cut-off value for hydrogen bond formation was limited to 3.5Å with an angle cut off 135°.

### Appendix A.2. Parameters Used during WaterSwap Analysis

Charge method implemented was Gasteiger or AM1-BCC, Solvate Box Buffer of 10.0 Angstrom, Minimize Energy Tolerance of 0.25 kcal/mol, Minimize Max Iterations sets the maximum number of iterations to perform for the minimization step was 1000, Equilibrate for 1 ns and Water Monitor Distance was selected of 7.00 Angstrom.

### Appendix A.3. WaterMap Parameters

Ligand was picked. Water was analyzed within 10.0 A of selected atoms (must be 5.0 A or more). Force field OPLS3e was selected. Treatment of existing waters as solvent. Simulation time was selected for 2.0 ns (use at least 1.5 ns).
