# Supplementary material for: Computational Design of Novel Allosteric Inhibitors for Plasmodium falciparum DegP
Source: Molecules. 2021 May 7;26(9):2742. doi: 10.3390/molecules26092742 (PMC8141111; doi:10.3390/molecules26092742)
Supplement: Supplementary file 1 [file molecules-26-02742-s001.zip › molecules-1144236-supplementary.pdf]

Computational design of novel allosteric inhibitors for *Plasmodium falciparum* DegP

Sadaf Shehzad[1], Rajan Pandey[1], Pawan Malhotra[2], Dinesh Gupta[1*]

[1]*Translational Bioinformatics Group, International Centre for Genetic Engineering and Biotechnology, New Delhi, 110067*

[2]*Malaria Biology Group, International Centre for Genetic Engineering and Biotechnology, New Delhi, 110067*

[*]*For correspondence*

Dr Dinesh Gupta: dinesh@icgeb.res.in

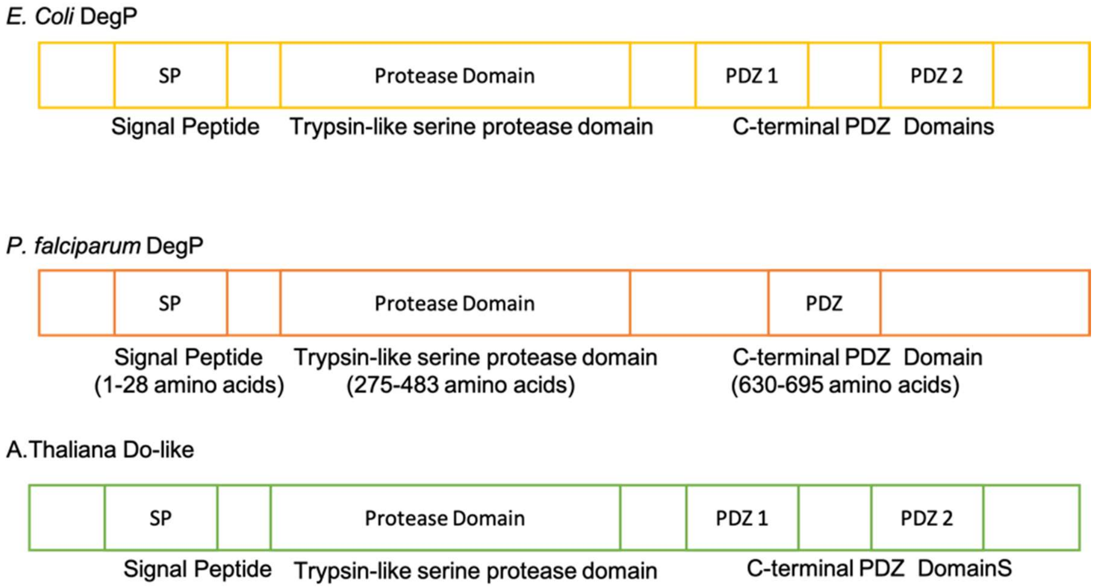

Figure S1: Identification of conserved domains of PfDegP by CDD predicting a signal sequence (1-28 amino acids), trypsin-like serine protease domain (275-483 amino acids) belonging to serine protease family and a PDZ domain (630 - 695 amino acids).

```
                                                   GSGF
Hs      ----------------ELFRKLPFSKRE----------VPVASGSGF-IVS--EDGLIVT    61
Ec      NFQQFFGDDSPFCQEGSPFQSSPFCQGGQGGNGGGQQQKFMALGSGVIIDA--DKGYVVT    102
Pf      --KYFKGVVKLYVDITEPNLEMIWQNYP----------PKSITGSGFIIEG----HLIIT    325
Pv      --KIYKGVVKLYVDITEPSLETIWSNSP----------PKRVSGSGFVIEG----GLILT    254
At      --LALNSVVKVFTVSSKPRLFQPWQITM----------QSESTGSGFVISG----KKILT    135
Tg      --EVPNTVFKVFCTHCEPNYSQPWTTRR----------QTTSMSTGFVTLDANGKQCLLT    323
                        .      :                   .:*.          ::*

          H
Hs      NAHVVTNKHRVKVEL-KNGATYEAKIKDVDEKADIALIKIDHQGKL----PVLLLGRSSE    116
Ec      NNHVVDNATVIKVQL-SDGRKFDAKMVGKDPRSDIALIQIQNPKNL----TAIKMADSDA    157
Pf      NAHNISYSTRILIRKHGNSGKYEAKILYVAHDVDIAILTTDDKTFFDDVY-ALHFGALPS    384
Pv      NAHNVAYSTRILVRKHGCSKKYEGAVLHVAHEADMALLTVADGSFYEDVS-ALELGPLPS    313
At      NAHVVANQTSVKVRKHGSTTKYKAKVQAVGHECDLAILEIDNDKFWEGMN-PLELGDIPS    194
Tg      NAHSVEHAAVVQVRKRGDHQKYEAEVLCIGLECDLAMLRVSDADFWKGLGPPLQWGPSPQ    383
        * * :     : :.      .:.. :       *:*::   .          :  .

                                                        D       S
Hs      LRPGEFVVAIGSPFSLQ-NTVTTGIVSTTQRGGKELGLRNSDMDYIQTDAIINYGNSGGP    175
Ec      LRVGDYTVAIGNPFGLG-ETVTSGIVSALGRSGLNA---ENYENFIQTDAAINRGNSGGA    213
Pf      LK--DEIITIGYPAGGDKLSVTEGIVSRIDVQYYKH--SNYKFLLTQIDAPLNPGNSGGP    440
Pv      LR--DDVITVGYPSGGDKLSVTKGIVSRIEVQYYRH--SNSRLLLTQIDAPMNPGNSGGP    369
At      MQ--DTVYVVGYPKGGDTISVSKGVVSRVGPIKYSH--SGTELLAIQIDAAINNGNSGGP    250
Tg      LG--DPVTVVGYPLGGDNSSVTQGVVSRADLQQYCL--GSCSLLAIQIDAAINPGNSGGP    439
        :   :   .:* * .    :*: *:**                   * **  :* *****

Hs      LVNLDGEVIGINTLKV-----TAGISFAIPSDKIKKFLTESHDRQAKGK-AITKK-----    224
Ec      LVNLNGELIGINTAILAPDGGNIGIGFAIPSNMVKNLTSQMVEYG------QVKR-----    262
Pf      ALVR-GKVVGICFQSY-KV--SNNISYIIPSTIISHFLLDIHKNKDYTGYAFLGVKYEPL    496
Pv      ALVK-GKVAGICFQLL-KM--ANNTSYIIPTPVIKHFLMDLHRSGKYNGYPSLGVKYLPL    425
At      VIMG-NKVAGVAFESL-CY--SDSIGYIIPTPVIRHFLNAIEESGEDVSFGSINLTYQKM    306
Tg      ALNRSSQCVGIAFQSL-KDGDTENIGYIIPSEVVSHFLEDYRRHGRCLGFGDGGFTWQKL    498
         :   .:  *:            .  .: **:  : ::

                          GLGF loop
Hs      ---------------------KYIGIRMMSLTSSKAKE-------LKDRHR-------DF    249
Ec      ---------------------GELGIMGTELNSELAKA-------M------------K     281
Pf      ENPSLREALGLEEMERKKIIKKNVGILITEVFEGHMSKQDDKYHDMDNKHHNVGDTHHNV    556
Pv      DNANLRRLLGLTDLERRREVEENSGILVTEVDEEQMGCQSGGGGSTKW---AVSSGEAST    482
At      DNDQLRKDFKMSDK--------MTGILINKINP---------------------------    331
Tg      ENKSLRHSLSLKSK--------DEGILIKKLDG---------------------------    523
                                **   .:
```

Figure S2: Multiple Sequence Alignment of PfDegP with selected organisms.

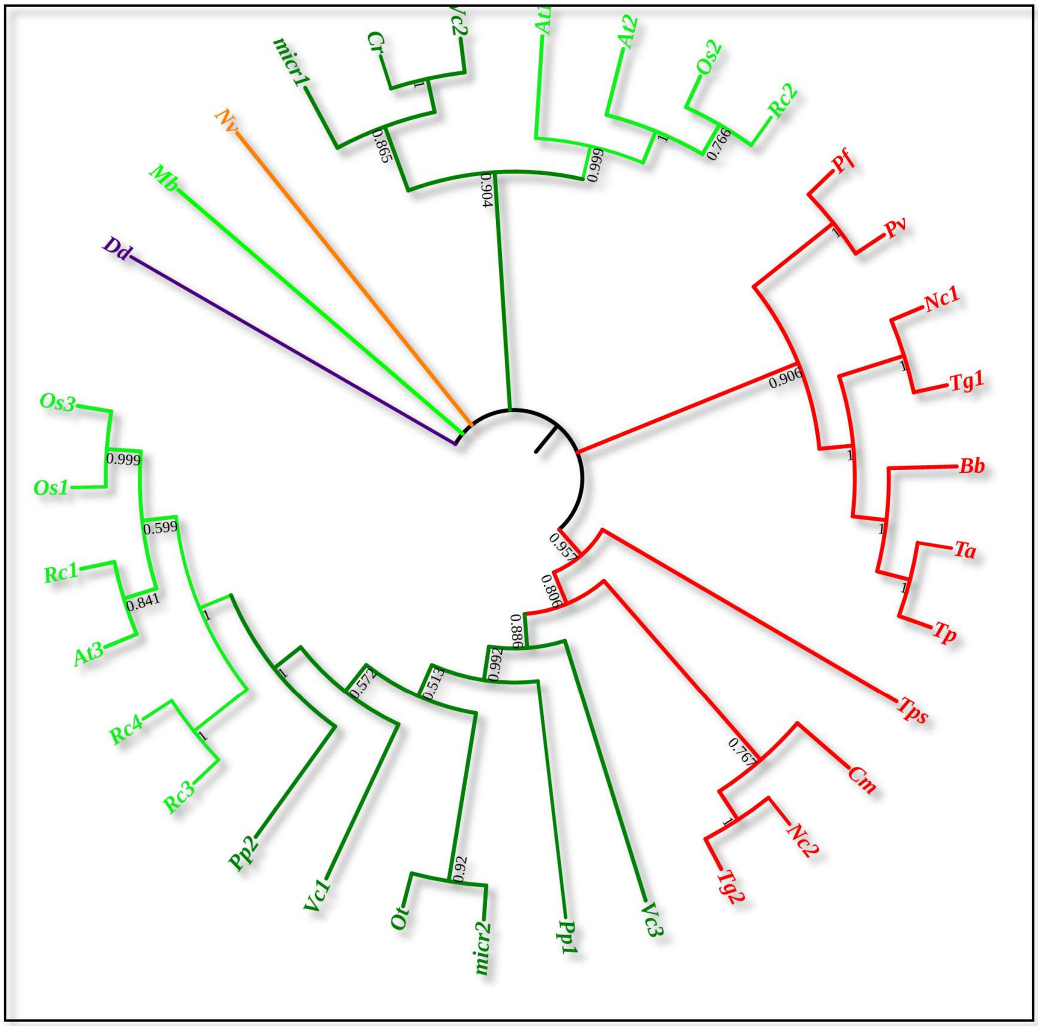

Figure S3: Phylogenetic tree of PfDegP: The evolutionary history was inferred using the Neighbor-Joining method. The bootstrap consensus tree inferred from 1000 replicates is taken to represent the evolutionary history of the taxa analyzed. Branches corresponding to partitions reproduced in less than 50% bootstrap replicates are collapsed. The evolutionary distances were computed using the JTT matrix-based method and are in the units of the number of amino acid substitutions per site. This analysis involved 33 amino acid sequences. All positions with less than 95% site coverage were eliminated, i.e., fewer than 5% alignment gaps, missing data, and ambiguous bases were allowed at any position (partial deletion option). There was a total of 438 positions in the final dataset. Evolutionary analyses were conducted in MEGA X. Plants are depicted in light green, algae and mosses in Dark green, apicomplexan parasites in Red, red algae as brown, sea anemone (orange) and amoeba depicted in purple color. Abbreviations used: *Dd- Dictyostelium discoideum, Mb- Monosiga brevicollis,Nv- Nematostella vectensis, micr1- Micromonas 1, Cr- Chlamydomonas reinhardtii, Vc2- Volvox carteri, At1-Arabidopsis thaliana 1, At2- Arabidopsis thaliana 2, Os2- Oryza sativa 2, Rc2- Ricinus communis 2, Pf- Plasmodium falciparum, Pv- Plasmodium vivax, Nc1- Neospora caninum 1, Tg1- Toxoplasma gondii 1, Bb- Babesia bovis, Ta- Theileria annulata, Tp- Theileria parva, Tps- Thalassiosira pseudonana, Cm- Cyanidioschyzon merolae, Nc2- Neospora caninum 2, Tg2- Toxoplasma gondii 2, Vc3- Volvox carteri 3, Pp1- Physcomitrella patens 1, micr2- Micromonas 2, Ot- Ostreococcus tauri, Vc1- Volvox carteri 1, Pp2- Physcomitrella patens 2, Rc3- Ricinus communis 3, Rc4- Ricinus communis 4, At3- Arabidopsis thaliana 3, Rc1- Ricinus communis 1, Os1- Oryza sativa 1, Os3- Oryza sativa 3.*

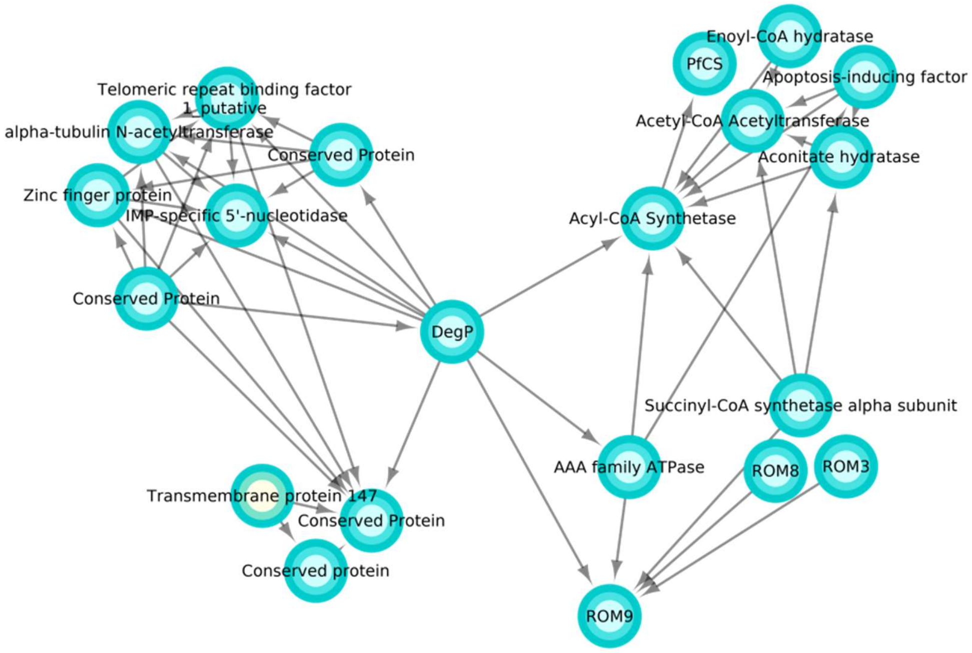

Figure S4: Protein-protein interactions of PfDegP predicted by STRING v11.0 and visualized using Cytoscape v 3.7 and network analyzer. The interactome reveals that PfDegP interacts with nucleotide synthesis pathway in parasite by interacting with IMP-specific 5'nucleotidase, in maintaining redox potential and apoptosis by regulating homeostasis by interacting with Acyl-CoA Synthetase

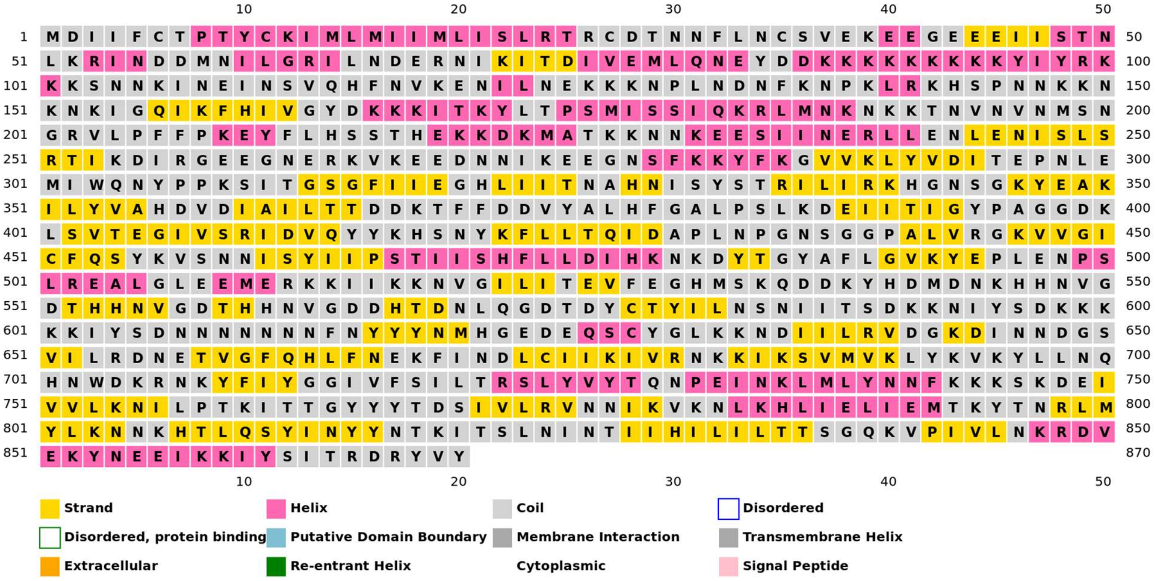

Figure S5: Secondary structure prediction of PfDegP using PSI-PRED

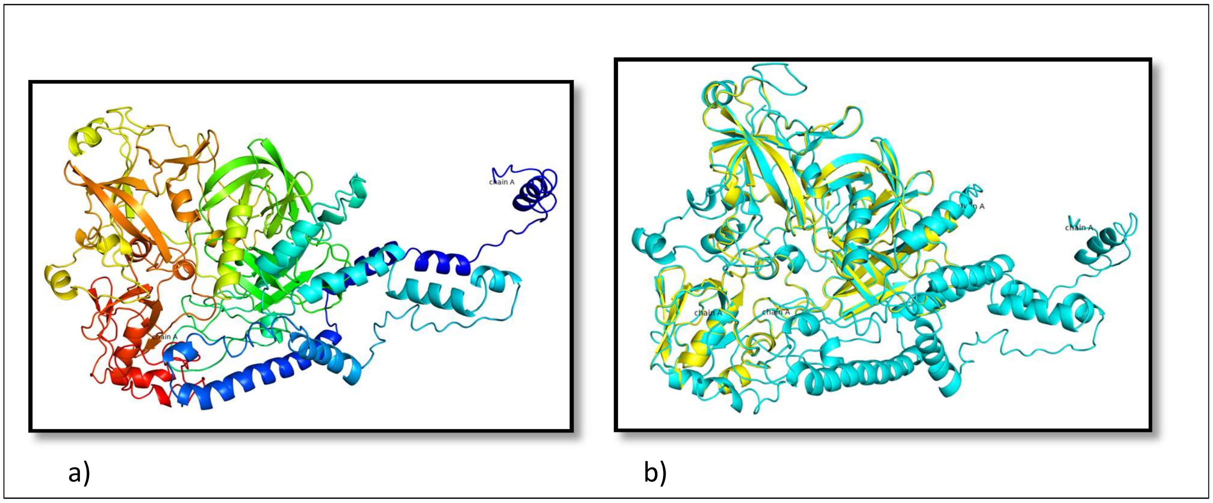

Figure S6: a) Predicted 3D structure of PfDegP using the template 4FLN_A (*Arabidopsis thaliana*, chloroplastic Protease Do-like 2), b) Superimposition of PfDegP homology model with the template (RMSD value of 0.203).

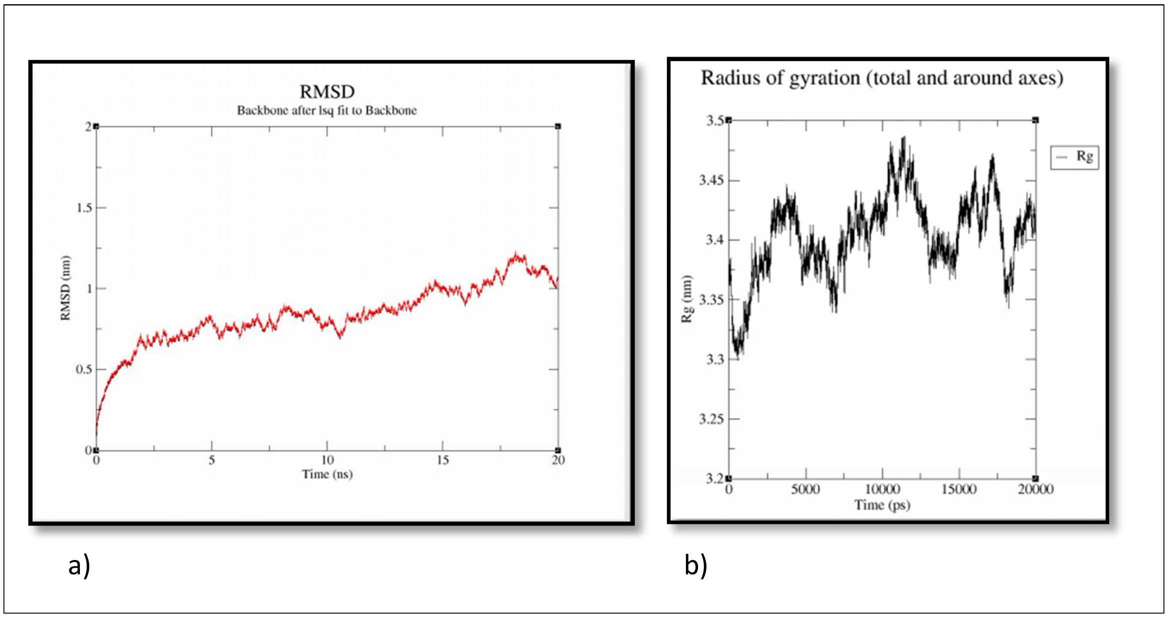

Figure S7: a) RMSD plot of PfDegP trajectories during molecular dynamics simulation of 10 ns b) Radius of gyration of PfDegP.

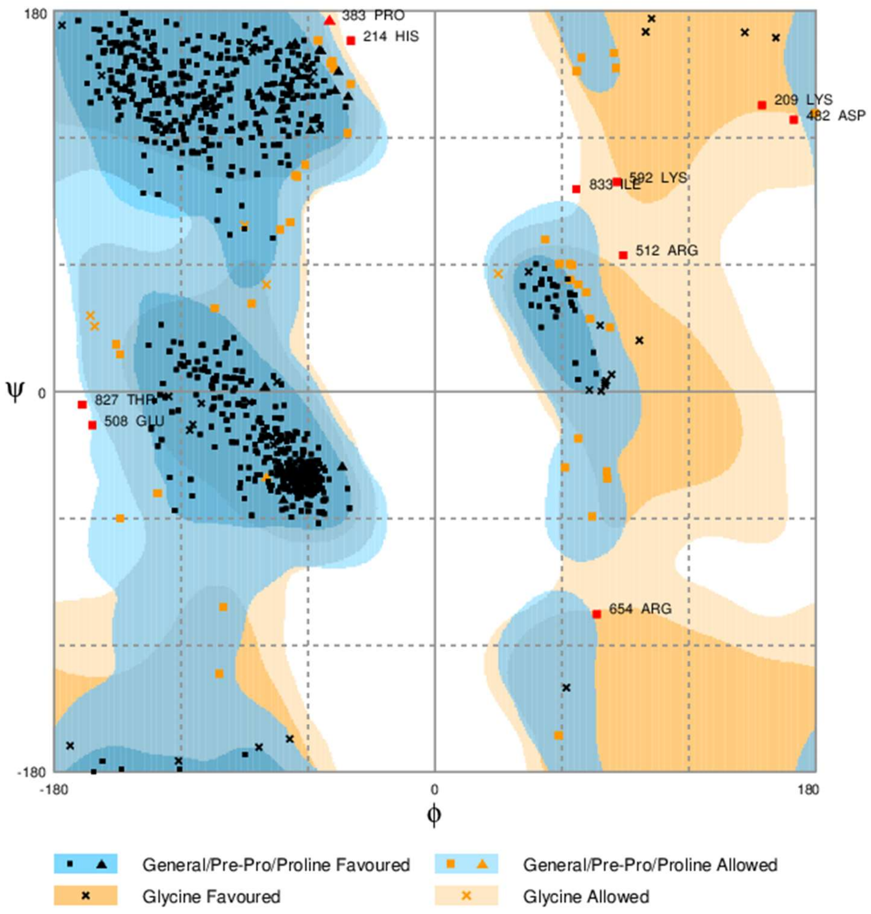

Number of residues in favoured region (~98.0% expected) : 811 (93.5%)
Number of residues in allowed region (~2.0% expected) : 46 (5.3%)
Number of residues in outlier region : 10 (1.2%)

Figure S8: Ramachandran Plot of PfDegP after simulation of 20 nanosecond.

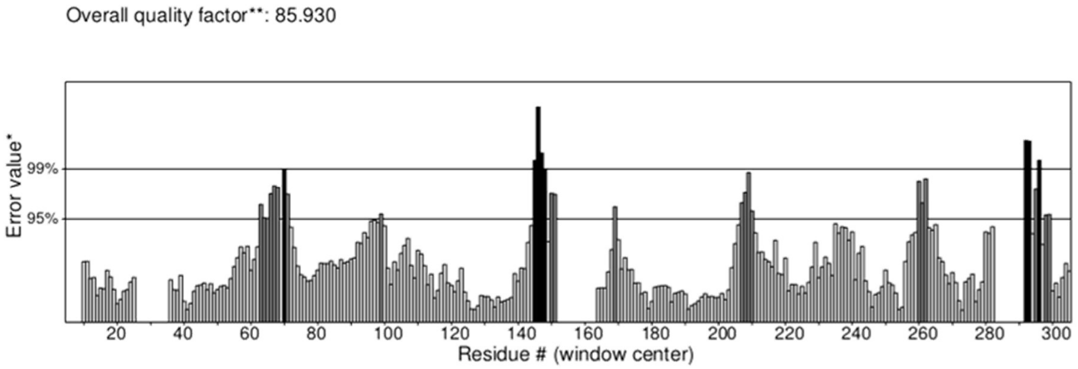

Figure S9: ERRAT score of the PfDegP model

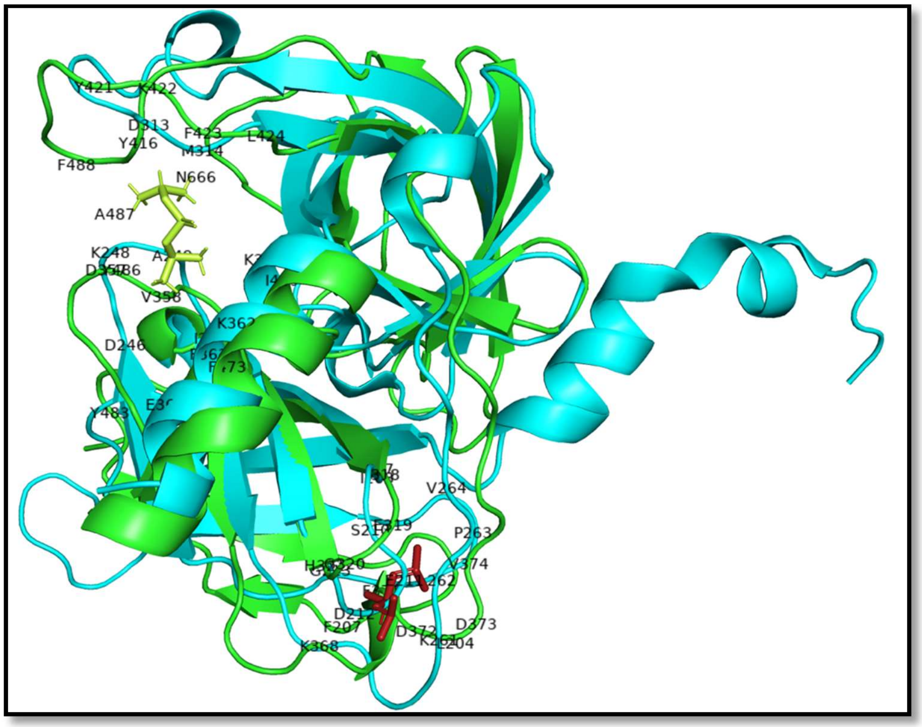

Figure S10: PfDegP (green in color) in docked pose with DFP (brick redin color) and human DegP, 3NZI (cyan in color) in docked pose with DFP (limon in color) exhibiting binding interactions in different pockets. The pocket residues are labelled in both 3NZI and PfDegP.

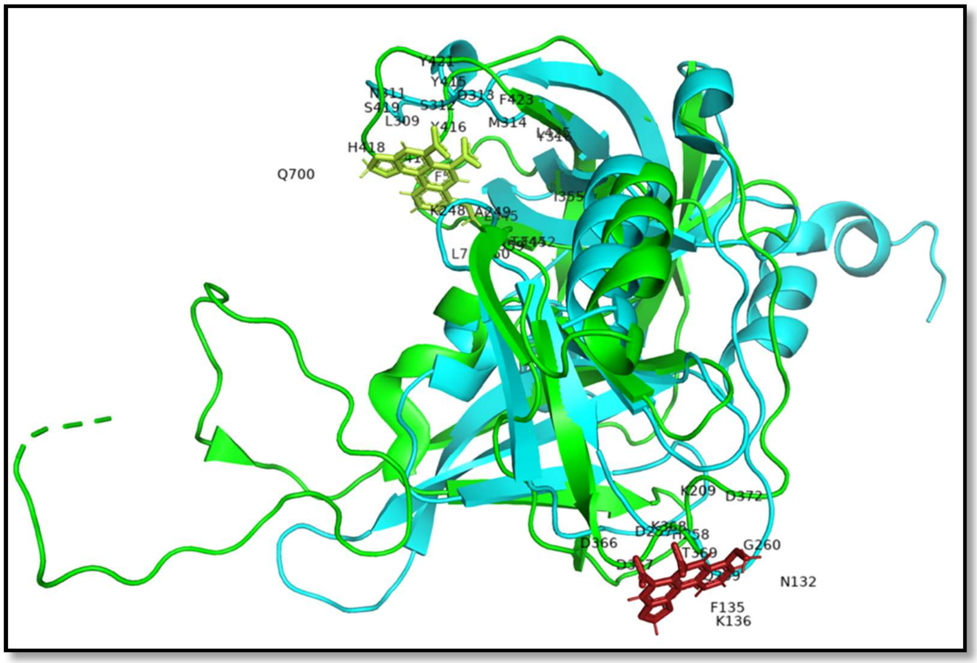

Figure S11: PfDegP (green in color) in docked pose with t2801 (brick red in color) and human DegP, 3NZI (cyan in color) in docked pose with t2801 (limon in color) exhibiting binding interactions in different pockets. The pocket residues are labelled in both 3NZI and PfDegP.

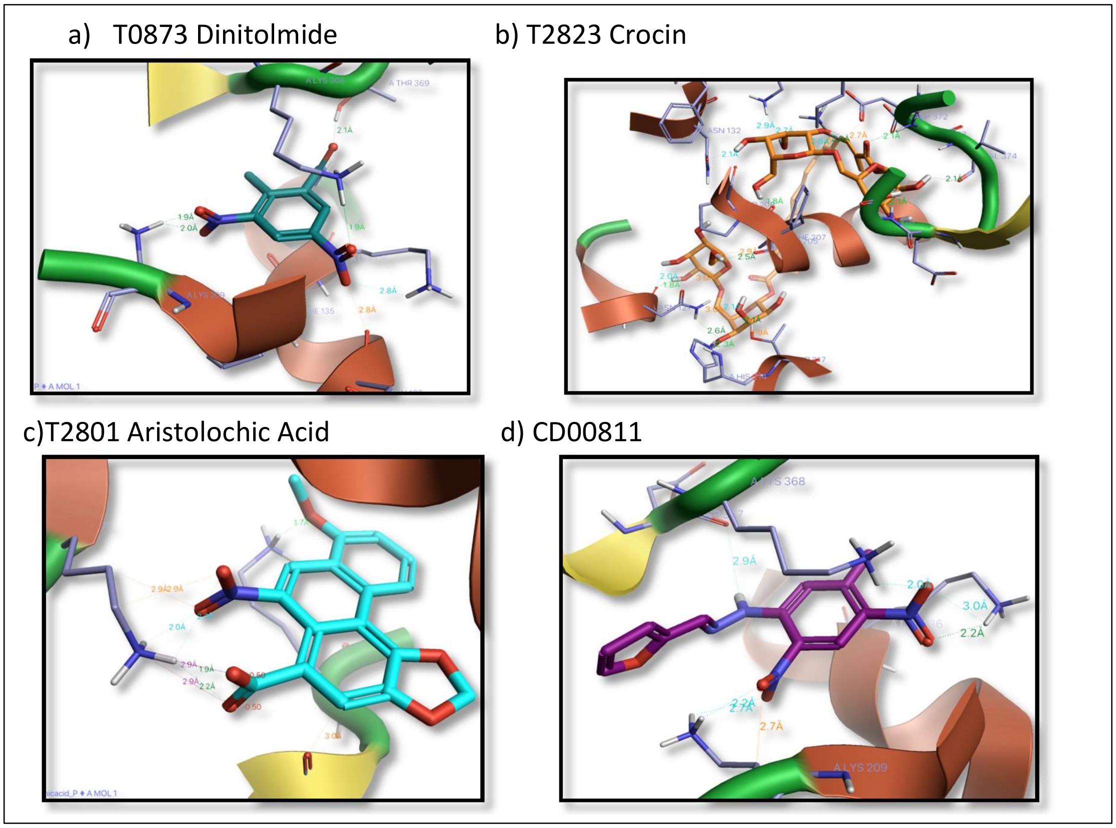

Figure S12: Showing interaction of potential hits of allosteric site binders: a) t0873 Dinitolmide, b) t2823 Crocin, c) t2801 Aristolochic Acid, d) CD00811 showing the residues involved in Hydrogen bonding and hydrophobic interactions.

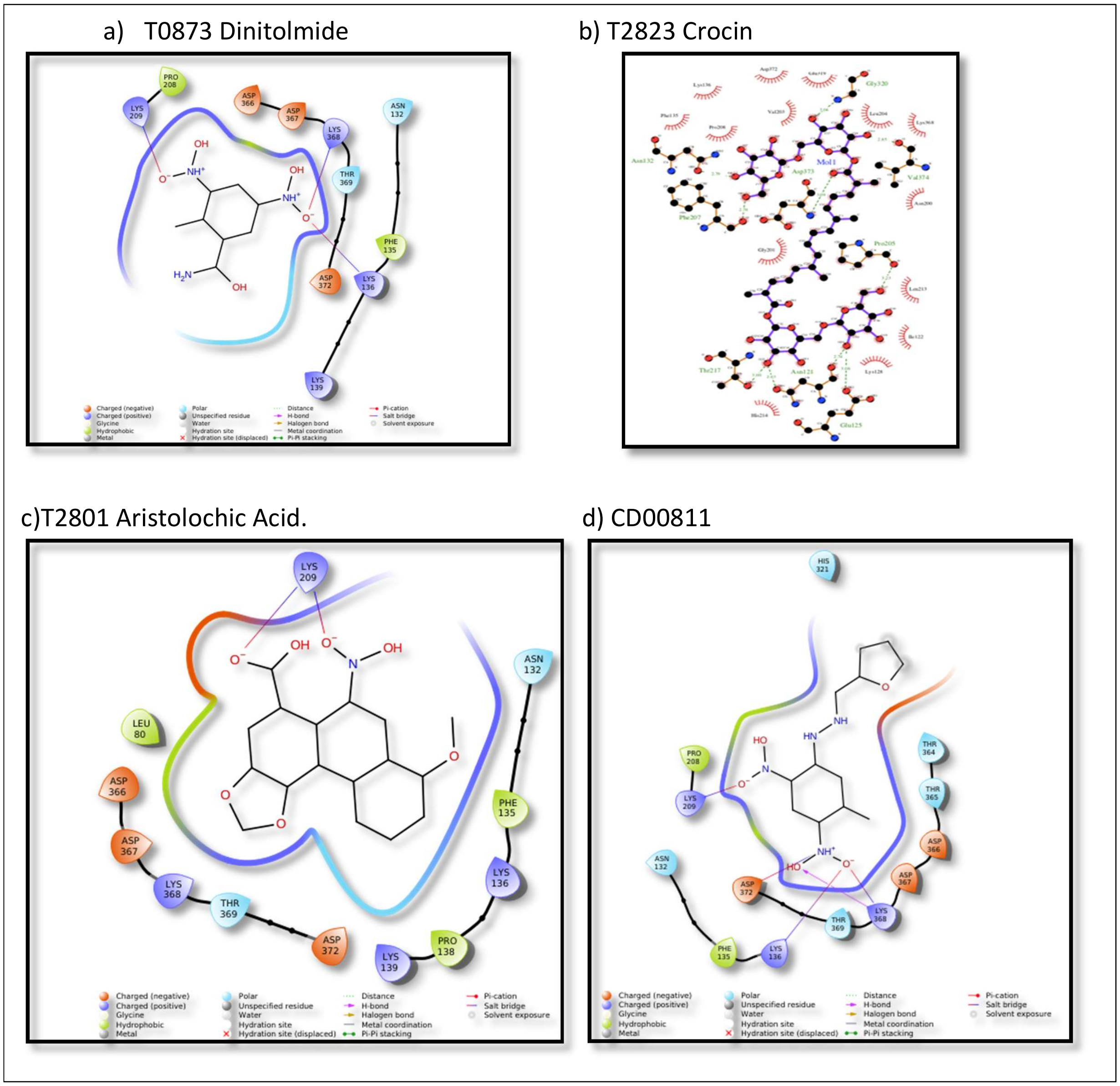

Figure S13: Ligand interaction diagram of a) t0873 Dinitolmide, b) t2823 Crocin, c) t2801 Aristolochic Acid, d) CD00811 showing the residues involved in Hydrogen bonding and hydrophobic interactions.

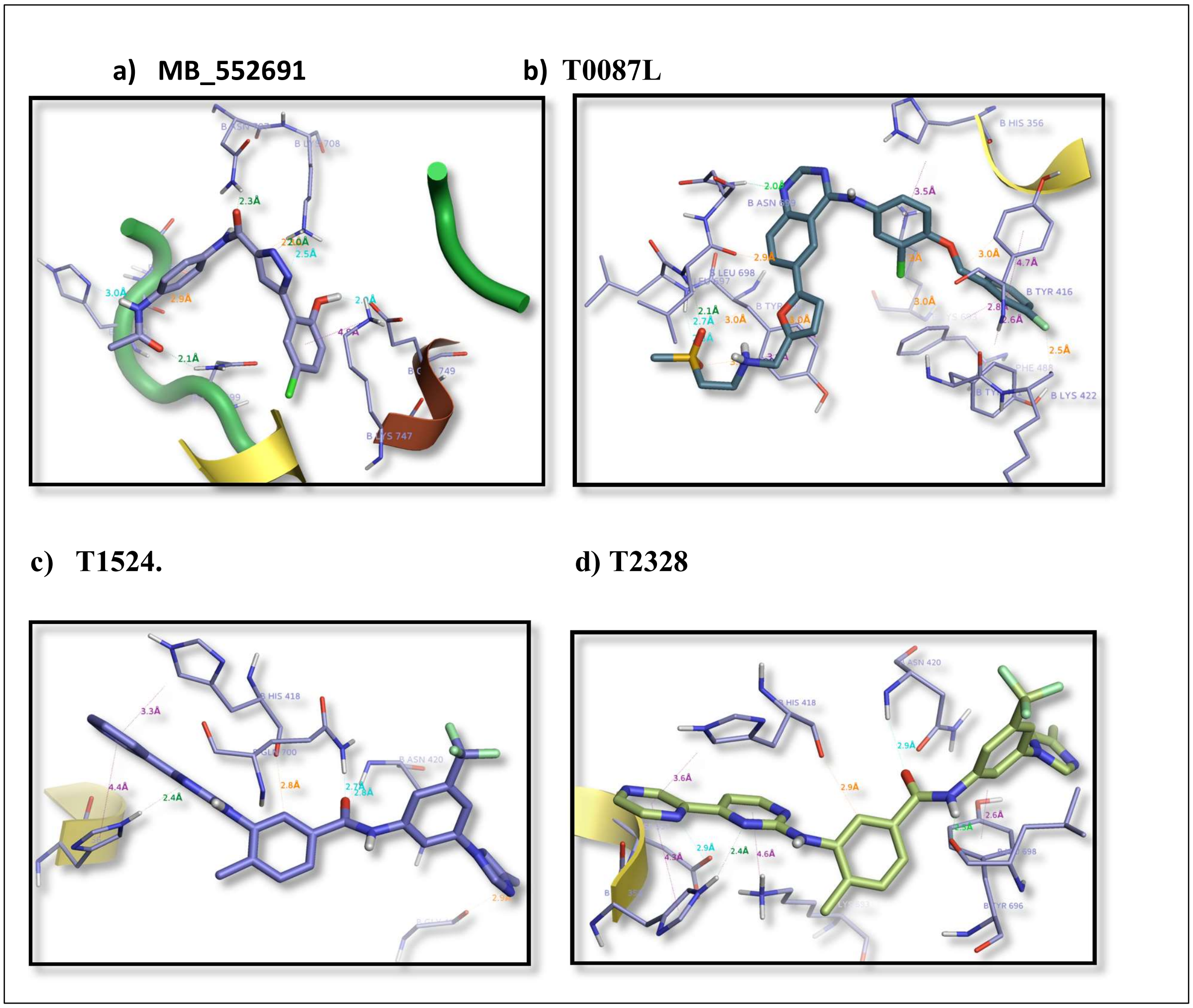

Figure S14: Showing interaction of potential hits: a) MB_552691, b) T0087L, c) T1524, d) T2328, showing the residues involved in hydrogen bonding and hydrophobic interactions.

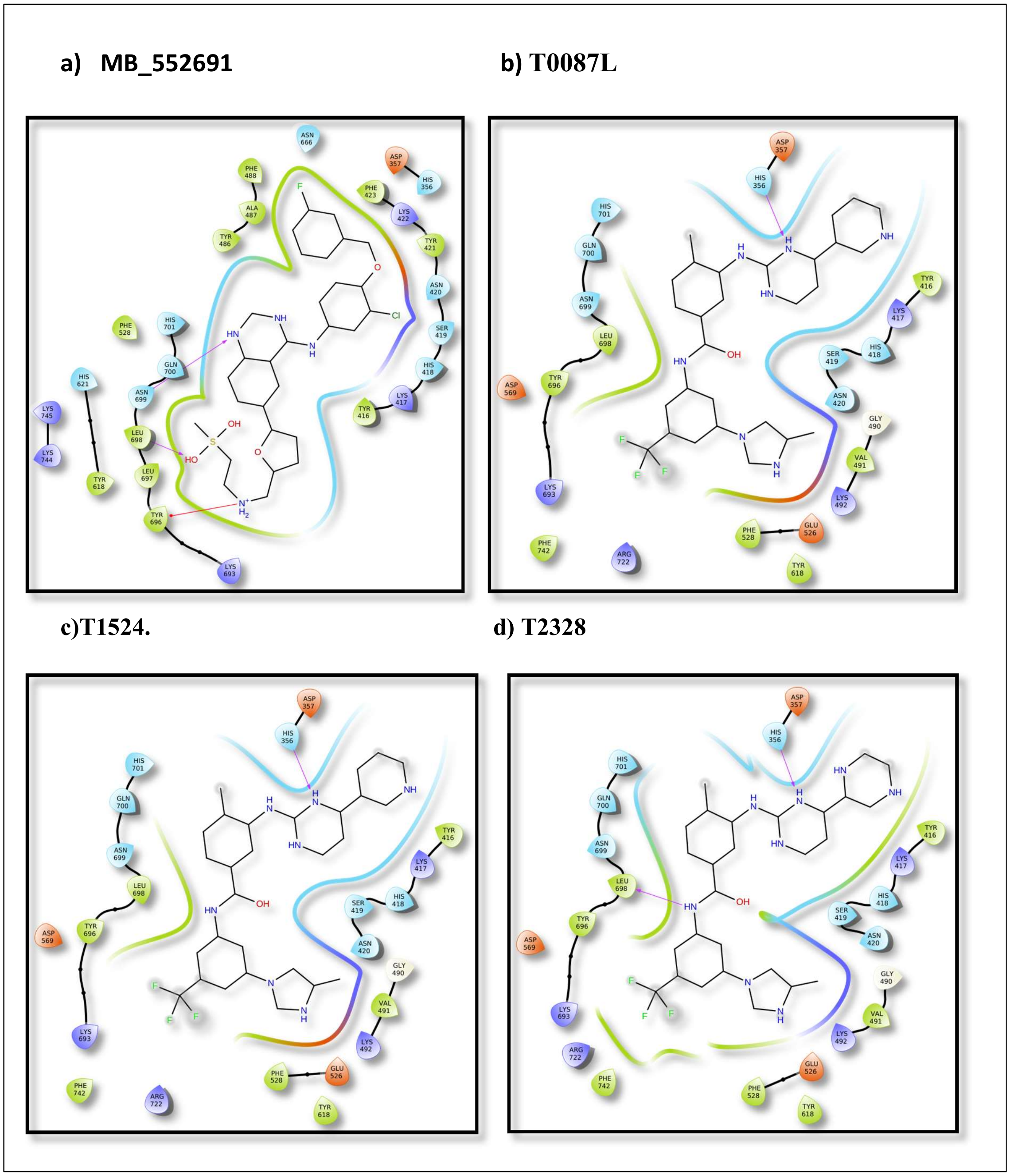

Figure S15: Ligand interaction diagrams: a) MB_552691, b) T0087L, c) T1524, d) T2328 showing the residues involved in Hydrogen bonding and hydrophobic interactions

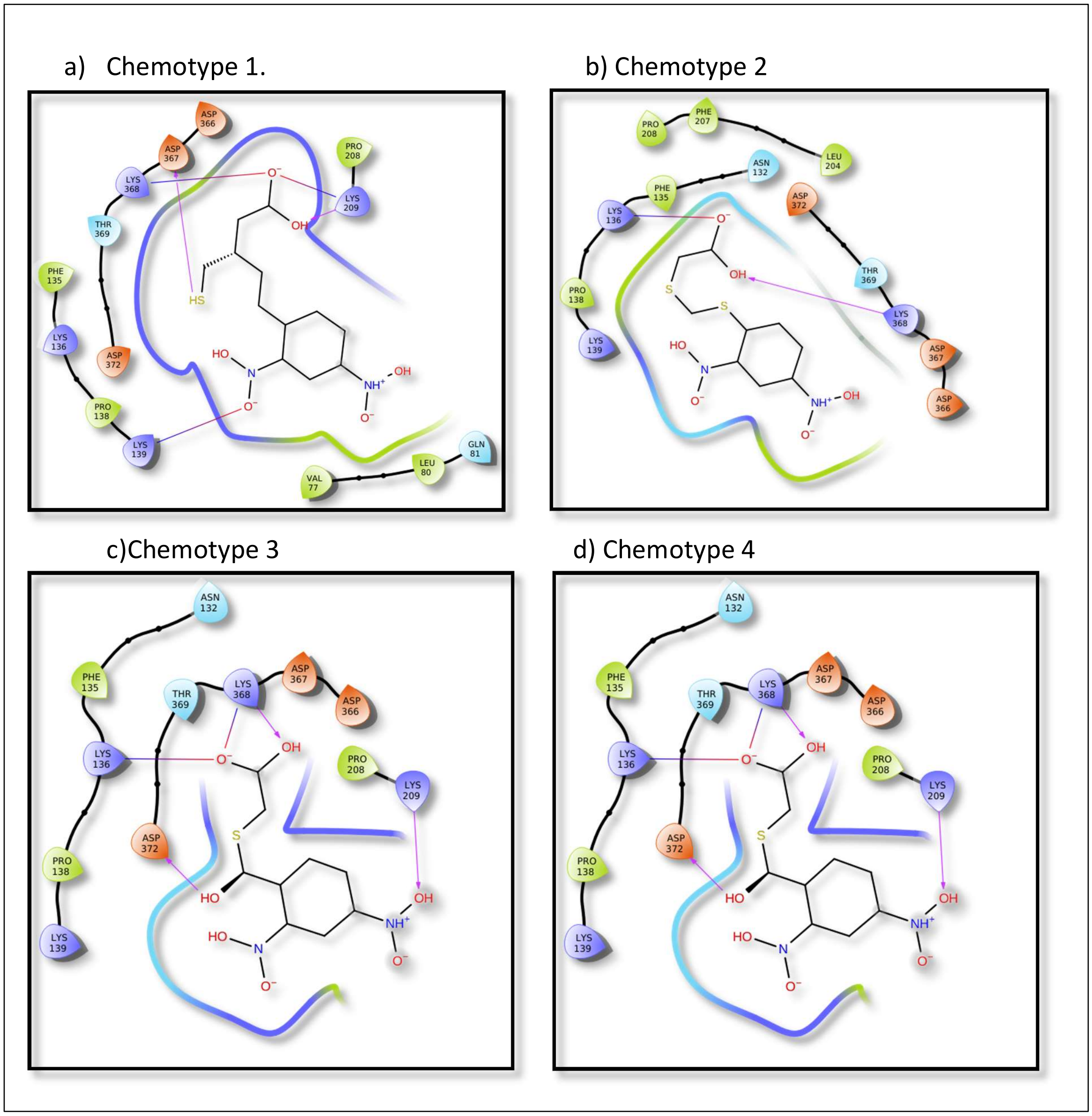

Figure S16: Interaction diagram of a) chemotype 1, b) chemotype 2, c) chemotype 3, d) chemotype 4, e) chemotype 5 showing the residues involved in Hydrogen bonding and hydrophobic interactions.

Table S1: The orthologs of PfDegP retrieved from OrthMCLDB having the orthoMCL ID OG5_133046.

S. No.	Taxonomy Name	Accession	Length	Product	Molecular Weight	PFam Domains
1	*Arabidopsis thaliana*	atha|NP_564856	559	DegP3 (DegP protease 3)	61713	PF00089
2	*Arabidopsis thaliana*	atha|NP_568543	586	DegP10 (DegP protease 10)	64727	PF00089
3	*Arabidopsis thaliana*	atha|NP_568577	592	DegP9 (DegP protease 9)	65153	PF00089
4	*Babesia bovis* T2Bo	bbov|XP_001610362.1	536	DegP protease	59911	PF00089
5	*Cyanidioschyzon merolae* strain 10D	cmer|CMM292C	596	null	66615	null
6	*Chlamydomonas reinhardtii*	crei|196391	812	Chlre3	84157	PF00089
7	*Dictyostelium discoideum* AX4	ddis|DDB_G0281081	648	null	72171	PF00089

8	*Monosiga brevicollis* MX1	mbre|fgenesh2_pg.scaffold_25000031	665	null	72269	PF00089
9	*Micromonas sp.* RCC299	micr|ACO61255	476	predicted protein	51550	PF00089
10	*Micromonas sp.* RCC299	micr|ACO63345	463	predicted protein	50883	PF00089
11	*Neospora caninum*	ncan|NCLIV_042710	952	Peptidase S1, chymotrypsin:PDZ/DHR/GLGF domain (Precursor), related	102961	null
12	*Neospora caninum*	ncan|NCLIV_068150	730	hypothetical protein	79959	PF00089
13	*Nematostella vectensis*	nvec|fgenesh1_pg.scaffold_67000052	570	null	63623	PF00089
14	*Oryza sativa* Japonica Group	osat|NP_001048089	567	Os02g0742500	61643	PF00089
15	*Oryza sativa* Japonica Group	osat|NP_001055560	614	Os05g0417100	67194	PF00089
16	*Oryza sativa* Japonica Group	osat|NP_001057235	628	Os06g0234100	68029	PF00089

17	*Ostreococcus tauri*	otau|e_gw1.05.00.88.1	546	null	59619	PF00089
18	*Plasmodium falciparum* 3D7	pfal|MAL8P1.126	870	serine protease, putative	101495	PF00089
19	*Physcomitrella patens* subsp. patens	ppat|estExt_fgenesh1_pg.C_80074	852	null	94760	PF00089
20	*Physcomitrella patens* subsp. patens	ppat|estExt_gwp_gw1.C_1760017	468	null	51659	PF00089
21	*Plasmodium vivax* SaI-1	pviv|PVX_088155	809	DegP-like serine protease 1 precursor, putative	87814	PF00089
22	*Ricinus communis*	rcom|29822.m003378	582	serine	64355	PF00089
23	*Ricinus communis*	rcom|29929.m004498	527	serine	59671	null
24	*Ricinus communis*	rcom|30147.m013987	524	serine	57700	PF00089
25	*Ricinus communis*	rcom|30147.m013988	569	serine	62552	PF00089

Table S2: Table depicting the percent identity and query coverage in model organisms. The model organisms viz., *Homo sapiens, Rattus rattus, Mus musculus, Equus caballus, Gallus gallus, E. coli* exhibited the percent identity between 28.39% to 34.38% and the query coverage ranging from 20 to 26% only. However, the orthologs considered by OrthoMCL were found to have an average of 44.7%.

26	*Theileria annulata* strain Ankara	tann|TA21265	576	serine protease (zymogen-like), putative	65340	PF00089
27	*Toxoplasma gondii*	tgon|TGME49_077850	744	trypsin, putative	81768	PF00089, PF00595
28	*Toxoplasma gondii*	tgon|TGME49_090840	960	trypsin, putative	102741	PF00089
29	*Theileria parva* strain Muguga	tpar|XP_765845	438	serine protease	49855	PF00089
30	*Thalassiosira pseudonana* CCMP1335	tpse|e_gw1.1.144.1	493	null	54102	PF00089
31	*Volvox carteri* f. nagariensis	vcar|XP_002948745	494	hypothetical protein VOLCADRAFT_58517	54361	PF00089
32	*Volvox carteri* f. nagariensis	vcar|XP_002951860	571	trypsin family	62925	PF00089
33	*Volvox carteri* f. nagariensis	vcar|XP_002958880	509	serine protease [*Volvox carteri f. nagariensis*]	54467	PF00089

**S. NO**	**Organism**	**Accession**	**Per. Identity**	**Query coverage**
1.	*Homo sapiens*	3NZI_A	33.02%	22%

2.	*Rattus rattus*	XP_032774748	33.06%	26%
3.	*Mus musculus*	NP_001074656.1	32.24%	26%
4.	*Equus caballus*	XP_023486546.1	31.98%	26%
5.	*Gallus gallus*	XP_015144381.1	34.38%	20%
6.	*E. coli*	WP_047400932.1	28.39%	25%

Table S3: The non PfDegP related biological activities of the shortlisted drug molecules, binding to PfDegP allosteric site

Structure	2D Structure	Title	Therapeutic use	Activity	Activity value (μm)	Bioassay name (ID)	Pubchem Link
[O-][N+](=O)c1cc([N+]([O-])=O)ccc1NCCCC([O-])=O	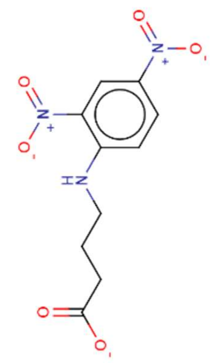	RJC02337	NA	NA	NA	NA	NA
O=C(N)c1cc([N+]([O-])=O)cc([N+]([O-])=O)c1C	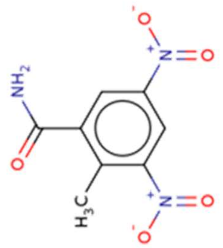	t0873	Coccidiostat: Dinitolmide exerts its/ greatest coccidiostatic activity against the asexual stages by arresting parasite development. Efficacy is limited to Eimeria tenell and E. necatrix unless combined with other products [1]	active	4.8966	qHTS assay to identify small molecules antagonists of the TGF-beta/Smad signalling pathway-cell viability counter screen (1346824)	https://pubchem.ncbi.nlm.nih.gov/compound/3092
O[C@@H]1[C@H](O[C@@H]([C@H](O)[C@@H]1O)CO)OC[C@@H]2[C@H](O)[C@H](O)[C@H](O)[C@H](O2)OC(=O)/C(=C/C=C/C(C)=C/C=C/C=C(\C=C\C=C(\C(O[C@@H]3[C@@H](O)[C@@H](O)[C@@H](O)[C@H](O3)CO[C@@H]4[C@@H](O)	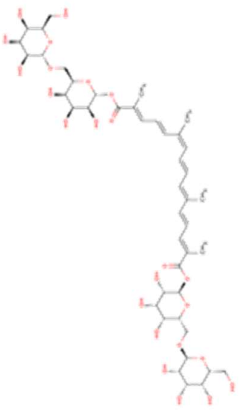	t2823	Crocin has been investigated for the treatment of Hyperglycemia, Metabolic Syndrome, Hypertriglyceridemi, and Hypercholesterolemia. cisplatin-induced hepatotoxicity via TLR4/NF-κBp50 signaling and BAMBI modulation of TGF-β activity: Involvement of miRNA-9 and miRNA-29 [2]	Active	17.7828	qHTS Assay for Inhibitors of Histone Lysine Methyltransferase G9a (504332)	https://pubchem.ncbi.nlm.nih.gov/compound/5281233#section=BioAssay-Results

[C@@H](O)[C@@H](O)[C@H](O4)CO)=O)C)C)C							
[O-][N+](=O)c1cc2c(OC)cccc2c3c4c(OCO4)cc(c13)C([O-])=O	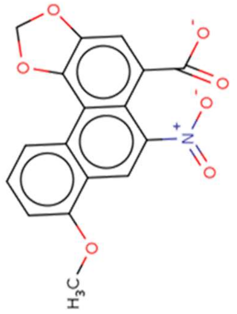	t2801	found in almost all Aristolochia (birthworts or pipevines) species. It has been tried in a number of treatments for inflammatory disorders, mainly in Chinese and folk medicine. It has a role as a nephrotoxin, a carcinogenic agent, a mutagen, a toxin and a metabolite [3]–[7]	Active	0.883	Growth inhibitory activity against human cancer cell line in the NCI's anticancer drug screening program (247402)	https://pubchem.ncbi.nlm.nih.gov/compound/2236
[O-][N+](=O)c1c(cc(N/N=C/c2ccco2)c([N+]([O-])=O)c1)C	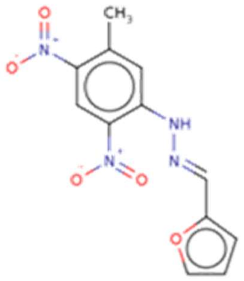	CD00811	NA	Inactive	NA	Screen for inhibitors of RMI FANCM (MM2) interaction (1159607)	https://pubchem.ncbi.nlm.nih.gov/compound/5713387
OP(OC(C)C)OC(C)C	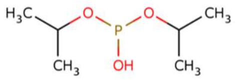	DFP	is used as ocular drops in the treatment of chronic glaucoma, acts as an irreversible cholinesterase inhibitor [8], [9]	Active	0.05	Inhibition of porcine liver carboxylesterase using [14C]GS-7340 substrate (279735)	https://pubchem.ncbi.nlm.nih.gov/compound/5936

Table S4: The drug activity towards other biological targets of the compounds identified as PfDegP inhibitors binding at catalytic site.

Structure	2D Structure	Title	Therapeutic use	Activity	Activity value (µm)	Bioassay name	Reference
O[C@H]1[C@H]2C=CC=C[C@H]2[S@]3(N1C)[C@H]4CCC[C@H]5CCC[C@@H]([C@H]54)[C@@H](O3)O	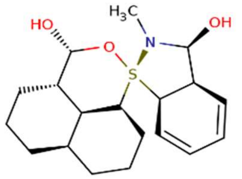	BTB11534	NA	NA	NA	NA	NA
Clc1ccc(O)c([C@H]2C[C@H](NN2)[C@@H](O)Nc3ccc(NC(=O)C)cc3)c1	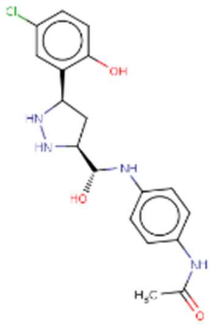	MB_552691	NA	NA	NA	NA	NA
Clc1cc(Nc2c3c(ncn2)ccc(c4ccc(o4)C[NH2+]CCS(=O)(=O)C)c3)ccc1OCc5cc(F)ccc5	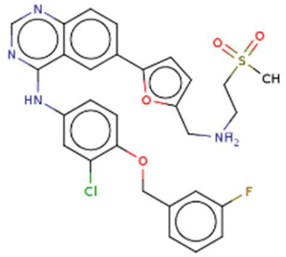	T0078L	Antineoplastic Agents; inhibitor of several Protein tyrosine kinase involved in tumor cell growth that is used in the therapy of advanced breast cancer and other solid tumors [10]–[12]	Active	0.00092	Kinase Inhibitor Selectivity Profiling Assay (1433)	https://pubchem.ncbi.nlm.nih.gov/compound/208908
FC(F)(F)c1cc(NC(=O)c2ccc(c(Nc3ncc(n3)-c4cnccc4)c2)C)cc(-n5cnc(c5)C)c1	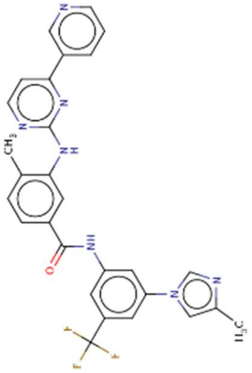	T1524	indicated for the treatment of:adult and paediatric patients with newly diagnosed Philadelphia chromosome positive chronic myelogenous leukaemia (CML) in the chronic phase,paediatric patients with Philadelphia chromosome positive CML in chronic phase with resistance or intolerance to prior therapy including imatinib [13]–[16]	Active	0.0001445	SANGER: Inhibition of human EoL-1-cell cell growth in a cell viability assay (742166).	https://pubchem.ncbi.nlm.nih.gov/compound/644241

FC(F)(F)c1cc(NC(=O)c2ccc(c(Nc3ncc(n3)-c4cnccn4)c2)C)cc(-n5cnc(c5)C)c1	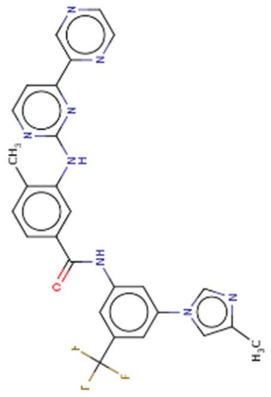	T2328	Radotinib is indicated for the treatment of different types of cancer, most notably Philadelphia chromosome-positive (Ph+) chronic myeloid leukemia (CML) with resistance or intolerance of other Bcr-Abl tyrosine-kinase inhibitors, such as patients resistant or intolerant to imatinib [17]–[19]	active	2.3919	Cytochrome P450 family 3 subfamily A member 4 (CYP3A4) small molecule antagonists: luciferase cell-based qHTS assay (1645841)	https://pubchem.ncbi.nlm.nih.gov/compound/16063245

Table S5: MMGBSA results of PfDegP-2236 complex.

Calculations performed using 50 complex frames.<br>Generalized Born ESURF calculated using 'LCPO' surface areas.<br>All units are reported in kcal/mole.				
GENERALIZED BORN:				
Complex:	Energy Component	Average	Std. Dev	Std. Err. of Mean
	VDWAALS	-6119.7052	32.4264	4.5858
	EEL	-60050.2088	291.2627	41.1908
	EGB	-18942.3451	206.7482	29.2386
	ESURF	414.3424	2.6950	0.3811
	G gas	-66169.9140	292.4279	41.3556
	G solv	-18528.0027	205.5169	29.0645
	TOTAL	-84697.9167	112.6449	15.9304
Receptor:	VDWAALS	-6094.8049	32.7327	4.6291
	EEL	-59647.0954	282.4948	39.9508
	EGB	-19281.7206	201.5997	28.5105
	ESURF	414.9038	2.6045	0.3683
	G gas	-65741.9003	283.2350	40.0555
	G solv	-18866.8169	200.4894	28.3535
	TOTAL	-84608.7171	111.3256	15.7438
Ligand:	VDWAALS	0.0898	1.7241	0.2438
	EEL	-7.6328	4.1386	0.5853
	EGB	-72.8517	2.3861	0.3374
	ESURF	2.8931	0.0344	0.0049
	G gas	-7.5429	3.4599	0.4893
	G solv	-69.9586	2.3782	0.3363
	TOTAL	-77.5016	2.1212	0.3000
Differences (Complex - Receptor - Ligand):	VDWAALS	-24.9902	2.4788	0.3506
	EEL	-395.4806	23.2483	3.2878
	EGB	412.2273	20.6015	2.9135
	ESURF	-3.4545	0.2266	0.0320
	G gas	-420.4708	22.0526	3.1187
	G solv	408.7728	20.5755	2.9098
	TOTAL	-11.6980	2.6091	0.3690

References:

[1] D. Qu, W. Ma, Y. Ye, and J. Han, "Effect of dinitolmide intercalated into Montmorillonite on E. tenella infection in chickens," *Parasitol. Res.*, vol. 113, no. 3, pp. 1233–1238, Mar. 2014, doi: 10.1007/s00436-014-3762-7.

[2] L. H. Khedr, R. M. Rahmo, D. B. Farag, M. F. Schaalan, and H. M. El Magdoub, "Crocin attenuates cisplatin-induced hepatotoxicity via TLR4/NF-κBp50 signaling and BAMBI modulation of TGF-β activity: Involvement of miRNA-9 and miRNA-29," *Food Chem. Toxicol.*, vol. 140, p. 111307, Jun. 2020, doi: 10.1016/j.fct.2020.111307.

[3] Y. Cui *et al.*, "Untargeted LC-MS-based metabonomics revealed that aristolochic acid I induces testicular toxicity by inhibiting amino acids metabolism, glucose metabolism, β-oxidation of fatty acids and the TCA cycle in male mice," *Toxicol. Appl. Pharmacol.*, vol. 373, pp. 26–38, Jun. 2019, doi: 10.1016/j.taap.2019.04.014.

[4] R.-M. Kavasi *et al.*, "Contact allergen (PPD and DNCB)-induced keratinocyte sensitization is partly mediated through a low molecular weight hyaluronan (LMWHA)/TLR4/NF-κB signaling axis," *Toxicol. Appl. Pharmacol.*, vol. 377, p. 114632, Aug. 2019, doi: 10.1016/j.taap.2019.114632.

[5] C. Li *et al.*, "Potent Inhibitors of Organic Anion Transporters 1 and 3 From Natural Compounds and Their Protective Effect on Aristolochic Acid Nephropathy," *Toxicol. Sci.*, vol. 175, no. 2, pp. 279–291, Jun. 2020, doi: 10.1093/toxsci/kfaa033.

[6] M. Sborchia *et al.*, "The impact of p53 on aristolochic acid I-induced nephrotoxicity and DNA damage in vivo and in vitro," *Arch. Toxicol.*, vol. 93, no. 11, pp. 3345–3366, Nov. 2019, doi: 10.1007/s00204-019-02578-4.

[7] J. Ye *et al.*, "Aristolochic acid I aggravates renal injury by activating the C3a/C3aR complement system," *Toxicol. Lett.*, vol. 312, pp. 118–124, Sep. 2019, doi: 10.1016/j.toxlet.2019.04.027.

[8] B. Gupta, R. Sharma, N. Singh, K. Kuca, J. R. Acharya, and K. K. Ghosh, "In vitro reactivation kinetics of paraoxon- and DFP-inhibited electric eel AChE using mono- and bis-pyridinium oximes," *Arch. Toxicol.*, vol. 88, no. 2, pp. 381–390, Feb. 2014, doi: 10.1007/s00204-013-1136-z.

[9] G. F. Makhaeva *et al.*, "Further studies toward a mouse model for biochemical assessment of neuropathic potential of organophosphorus compounds: Mouse model for biochemical OPIDN assessment," *J. Appl. Toxicol.*, vol. 34, no. 12, pp. 1426–1435, Dec. 2014, doi: 10.1002/jat.2977.

[10] Y. Asari, K. Kageyama, A. Sugiyama, H. Kogawa, K. Niioka, and M. Daimon, "Lapatinib decreases the ACTH production and proliferation of corticotroph tumor cells," *Endocr. J.*, vol. 66, no. 6, pp. 515–522, 2019, doi: 10.1507/endocrj.EJ18-0491.

[11] S. A. Coker *et al.*, "The effects of lapatinib on cardiac repolarization: results from a placebo controlled, single sequence, crossover study in patients with advanced solid tumors," *Cancer Chemother. Pharmacol.*, vol. 84, no. 2, pp. 383–392, Aug. 2019, doi: 10.1007/s00280-019-03880-9.

[12] Y. Zhang, R. A. Greer, Y. Song, H. Praveen, and Y. Song, "In silico identification of available drugs targeting cell surface BiP to disrupt SARS-CoV-2 binding and replication: Drug repurposing approach," *Eur. J. Pharm. Sci.*, vol. 160, p. 105771, May 2021, doi: 10.1016/j.ejps.2021.105771.

[13] H. Kantarjian *et al.*, "Nilotinib in Imatinib-Resistant CML and Philadelphia Chromosome–Positive ALL," *N Engl J Med*, p. 10, 2006.

[14] M. Breccia, L. Cannella, M. Nanni, C. Stefanizzi, and G. Alimena, "Nilotinib Can Override Dasatinib Resistance in Chronic Myeloid Leukemia Patients with Secondary Resistance to Imatinib First-Line Therapy," *Acta Haematol.*, vol. 118, no. 3, pp. 162–164, 2007, doi: 10.1159/000108639.

[15] H. M. Kantarjian *et al.*, "Nilotinib (formerly AMN107), a highly selective BCR-ABL tyrosine kinase inhibitor, is effective in patients with Philadelphia chromosome–positive chronic myelogenous leukemia in chronic phase following imatinib resistance and intolerance," *Blood*, vol. 110, no. 10, pp. 3540–3546, Nov. 2007, doi: 10.1182/blood-2007-03-080689.

[16] T. Maekawa, E. Ashihara, and S. Kimura, "The Bcr-Abl tyrosine kinase inhibitor imatinib and promising new agents against Philadelphia chromosome-positive leukemias," *Int. J. Clin. Oncol.*, vol. 12, no. 5, pp. 327–340, Oct. 2007, doi: 10.1007/s10147-007-0699-1.

[17] S.-H. Kim *et al.*, "Efficacy and safety of radotinib in chronic phase chronic myeloid leukemia patients with resistance or intolerance to BCR-ABL1 tyrosine kinase inhibitors," *Haematologica*, vol. 99, no. 7, pp. 1191–1196, Jul. 2014, doi: 10.3324/haematol.2013.096776.

[18] T. O'Hare, M. S. Zabriskie, A. M. Eiring, and M. W. Deininger, "Pushing the limits of targeted therapy in chronic myeloid leukaemia," *Nat. Rev. Cancer*, vol. 12, no. 8, pp. 513–526, Aug. 2012, doi: 10.1038/nrc3317.
[19] M. S. Zabriskie, N. A. Vellore, K. C. Gantz, M. W. Deininger, and T. O'Hare, "Radotinib is an effective inhibitor of native and kinase domain-mutant BCR-ABL1," *Leukemia*, vol. 29, no. 9, pp. 1939–1942, Sep. 2015, doi: 10.1038/leu.2015.42.
